# Predicting the potential global distribution of *Ageratina adenophora* under current and future climate change scenarios

**DOI:** 10.1002/ece3.7974

**Published:** 2021-08-08

**Authors:** Gu Changjun, Tu Yanli, Liu Linshan, Wei Bo, Zhang Yili, Yu Haibin, Wang Xilong, Yangjin Zhuoga, Zhang Binghua, Cui Bohao

**Affiliations:** ^1^ Key Laboratory of Land Surface Pattern and Simulation Institute of Geographic Sciences and Natural Resources Research CAS Beijing China; ^2^ University of Chinese Academy of Sciences Beijing China; ^3^ Tibet Plateau Institute of Biology Lhasa China; ^4^ School of Life Sciences Guangzhou University Guangzhou China

**Keywords:** *Ageratina adenophora*, climate change, ecological niche modeling, invasive alien species, MaxEnt

## Abstract

**Aim:**

Invasive alien species (IAS) threaten ecosystems and humans worldwide, and future climate change may accelerate the expansion of IAS. Predicting the suitable areas of IAS can prevent their further expansion. *Ageratina adenophora* is an invasive weed over 30 countries in tropical and subtropical regions. However, the potential suitable areas of *A. adenophora* remain unclear along with its response to climate change. This study explored and mapped the current and future potential suitable areas of *Ageratina adenophora*.

**Location:**

Global.

**Taxa:**

Asteraceae *A. adenophora (Spreng.)* R.M.King & H.Rob. Commonly known as Crofton weed.

**Methods:**

Based on *A. adenophora* occurrence data and climate data, we predicted its suitable areas of this weed under current and future (four RCPs in 2050 and 2070) by MaxEnt model. We used ArcGIS 10.4 to explore the potential suitable area distribution characteristics of this weed and the “ecospat” package in R to analyze its altitudinal distribution changes.

**Results:**

The area under the curve (AUC) value (>0.9) and true skill statistics (TSS) value (>0.8) indicated excelled model performance. Among environment factors, mean temperature of coldest quarter contributed most to the model. Globally, the suitable areas for *A*. *adenophora* invasion decreased under climate change scenarios, although regional increases were observed, including in six biodiversity hotspot regions. The potential suitable areas of *A*. *adenophora* under climate change would expand in regions with higher elevation (3,000–3,500 m).

**Main conclusions:**

Mean temperature of coldest quarter was the most important variable influencing the potential suitable area of *A. Adenophora*. Under the background of a warming climate, the potential suitable area of *A*. *adenophora* will shrink globally but increase in six biodiversity hotspot regions. The potential suitable area of *A*. *adenophora* would expand at higher elevation (3,000–3,500 m) under climate change. Mountain ecosystems are of special concern as they are rich in biodiversity and sensitive to climate change, and increasing human activities provide more opportunities for IAS invasion.

## Graphical abstract

Invasive alien species (IAS) are a troublesome problem worldwide. IAS pose great threats to local ecosystems along with enormous economic loss. Unfortunately, we do not have effective ways to control the spread of most IAS. Predicting suitable distributions of IAS is an effective way to prevent their further expansion. *Ageratina adenophora* is a typical vicious invasive weed in more than 30 countries located in tropical and subtropical regions. However, little is known about its potential distribution globally and its response to future climate change. In this study, we attempted to address these gaps in knowledge. We hope that this study provides valuable reference information for preventing invasion by *A. adenophora*.

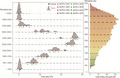

## 1 INTRODUCTION

Invasive alien species (IAS) are recognized as one of the main drivers of global environmental change (Simberloff et al., 2013). IAS lead to biodiversity loss (Bellard et al., 2016; Clavero & Garciaberthou, 2005), affect the ecosystem function and services (Vilà et al., 2010), and cause economic losses (Diagne et al., 2020; Ekesi et al., 2016; Paini et al., 2016). Climate change and anthropogenic activities, such as international trade, tourism, and road network expansion, play important roles in the expansion of IAS (Bertelsmeier et al., 2015, 2017; Wan & Wang, 2018) by providing opportunities for IAS to spread and accelerating IAS expansion (Wang, Wan, et al., 2017). IAS are commonly believed to be closely related to climate change (Alexander et al., 2016; Merow et al., 2017; Rodríguez‐Merino et al., 2018; Zhao et al., 2013), and Richardson and Rejmánek (2011) predicted that climate change will accelerate IAS invasion. However, the relationship between IAS and climate change remains unclear since their interaction is quite complex (Merow et al., 2017). Exploring the spatial patterns of potentially suitable areas for IAS at present and in future is an effective way to prevent the further expansion of IAS (Fournier et al., 2019; Kaiser & Burnett, 2010; Keller et al., 2007). Several recent studies have analyzed the potential changes in IAS distributions under multiple climate change scenarios at regional and global scales. Species distribution models (SDMs) have been widely applied in the early detection IAS (Ahmad et al., 2019; Padalia et al., 2014; Rodríguez‐Merino et al., 2018; Srivastava et al., 2018; Zhang et al., 2015; Zhao et al., 2013) by mapping potential IAS distribution and quantifying the relationships between IAS and environmental factors based on occurrence‐only data and species habitat conditions (e.g., climate, soil conditions, and terrain).

*Ageratina adenophora* (Sprengel) R. King and H. Robinson (synonym: *Eupatorium adenophorum* Sprengel), also known as Crofton weed, is regarded as one of the most serious invasive species in Asia, Africa, and Oceania (Tang et al., 2019). *A*. *adenophora* is native to Mexico (Qiang, 1998) and was introduced as an ornamental plant to other regions, including the United Kingdom (Auld & Martin, 1975), Hawaii (Muniappan et al., 2009), Australia (Auld, 1969), India (Bhatt et al., 2012; Poudel et al., 2019), South Africa (Kluge, 1991), Nepal (Tiwari, 2005), and Italy (Del Guacchio, 2013). *A*. *adenophora* is classified as one of the worst IAS in China (Yan et al., 2001; Zhang et al., 2008). The ecological attributes of *A*. *adenophora* contribute to its invasive ability. First, it possesses strong sexual and asexual reproductive capacity (Feng, 2008). According to Parsons (1992), one ramet can produce up to 10,000 seeds per season, including some 15% to 30% viable seeds. The seeds are capable of discontinuous germination, which prolongs their viability (Shen et al., 2011). Furthermore, the seeds are tiny scale, facilitating their spread by wind and water; the seeds of *A. adenophora* can disperse over both short and long distances (Wang et al., 2011; Zhang et al., 2008). *A. adenophora* also possesses a strong allelopathic effect, allowing it to compete with native species (Heather et al., 2011; Zhong et al., 2007). Research has shown that *A*. *adenophora* can alter the soil microbial community, which may inhibit native species and benefited its own growth (Niu, Liu, Wan, & Liu, 2007; Xu et al., 2012). In combination with the above traits, the high‐stress tolerance (Li et al., 2008; Rivera et al., 2017) and high morphological plasticity (Shen, 2019; Zhao et al., 2013) of *A. adenophora* make it an “ideal” weed (Baker et al., 1965). The invasion of *A*. *adenophora* has significantly influenced the native biodiversity and resulted in enormous economic losses (Hui et al., 2007; Xianming et al., 2013; Xu et al., 2006; Yu, Huang, et al., 2014). Various countermeasures against *A. adenophora* invasion have been implemented, including chemical control and biological control based on its invasion mechanism; however, no single control approach is effective (Yang et al., 2017).

Preventing the invasion of IAS into new potentially suitable regions is thought to be the most effective way of controlling the damage and costs to both the ecosystem and economy (Fournier et al., 2019). SDMs play an important role in risk assessment and conservation (Jiménez‐Valverde et al., 2011) as they can be used to investigate the relationships between species occurrence data and the background environmental conditions (Yue et al., 2019). Predictions can then be made based on these relationships (Galletti et al., 2013; Yang et al., 2013; Zhang et al., 2018). The prediction of potentially suitable areas for species makes it possible for policymakers to enact measures to prevent IAS invasion. Numerous modeling methods are available for prediction, including the generalized linear model (He, Chen, et al., 2019), evolutionary algorithms (Gobeyn et al., 2019), random forest (Fern et al., 2019), Bayesian hierarchical logistic mixed model (Rocchini et al., 2019), and the maximum entropy (MaxEnt) model (Phillips et al., 2017). Although it is difficult to identify the most appropriate method (Elith et al., 2010), MaxEnt was applied in this study because of demonstrated ability to predict species distributions and superior performance compared with other presence‐only SDMs (Abolmaali et al., 2018; Galletti et al., 2013; Qin et al., 2017; Tererai & Wood, 2014; Yi et al., 2016; Zhang et al., 2018).

This study aimed to address the following questions: (i) What are the potential spatial patterns of *A*. *adenophora* under current conditions and under different future climate change scenarios? (ii) Where are the high‐invasion‐risk regions at present and in the future? We hope that the findings of this study contribute to preventing the further invasion of *A*. *adenophora*.

## 2 MATERIALS AND METHODS

### 2.1 Environmental variables

For climate data, 19 bioclimatic variables were obtained from the WorldClim dataset (http://www.worldclim.org/), with a 1‐km spatial resolution (Hijmans et al., 2005). The WorldClim dataset has been widely used in species distribution modeling (He, Su, et al., 2019; Jiao et al., 2019; Tan et al., 2019; Yue et al., 2019). Two versions of the WorldClim dataset are available (version 2.0 and version 1.4). The dataset includes past and future (version 1.4 only) climate conditions at four different resolutions (10, 5, 2.5 min, and 30 s). Version 1.4 with a resolution of 30 s was selected for use in this study, and the average data for the years 1970–2000 were used to represent the current climate conditions. The climate projections in WorldClim come from the Fifth Assessment Report of the Intergovernmental Panel on Climate Change (IPCC) and have been downscaled and calibrated. In this study, we selected Climate Community Climate System Model version 4 (CCSM4) to represent the future climate scenarios (Gent et al., 2011). To indicate future climatic scenarios, we chose the data for 2050 and 2070 under four representative concentration pathways (RCPs): RCP2.6, RCP4.5, RCP6.0, and RCP8.5 (Ahmad et al., 2019). Soil data were downloaded from (http://soilgrids.org) at a resolution of 1 km, and 12 soil variables were selected to indicate the soil conditions (The PLOS One Staff, 2014). Terrain factors alter the redistribution of precipitation and solar radiation, resulting in mountain climate patterns. As previous research indicated that mountain ecosystems are more sensitive to climate change (Steinbauer et al., 2018), which is expected to trigger an upward expansion of plants in mountain regions (Grabherr et al., 1994; Walther et al., 2002). This finding has been proved that many native species have already shifted their distributions to a higher elevation (Chen et al., 2011; Lenoir et al., 2008). In addition, both habitat and elevation can restrict species ranges (Harris & Pimm, 2008; Sekercioglu et al., 2008) and have shown to be important in explaining the distribution of species (Luoto & Heikkinen, 2008; Virkkala et al., 2010). For the purpose of exploring the potential impacts of terrain factors, thus we added the topo covariate, including elevation, slope, and aspect (Manzoor et al., 2018). Terrain factors were derived from digital elevation model data, which were downloaded at (http://srtm.csi.cgiar.org/) and included elevation, slope, and aspect. We obtained land‐cover data at 1‐km resolution from the EarthEnv dataset (https://www.earthenv.org/landcover), which integrates multiple global land‐cover datasets (Tuanmu & Jetz, 2015). For many applications in biodiversity and ecology, existing remote sensing‐derived land‐cover products are limited by inconsistency issues and their typically noncontinuous nature. The consensus product with the generalized scheme better captures land‐cover heterogeneity and has improved utility for modeling species distributions. Two versions of the dataset are available: the full version and reduced version. The former dataset integrates GlobCover (2005‐06; v2.2), the MODIS land‐cover product (MCD12Q1; v051), GLC2000 (global product; v1.1), and DISCover (GLCC; v2); the latter only includes the first three datasets. In this study, we used the full version which includes 12 land‐cover classes. The values of each land‐cover class range from 0 to 100, representing the consensus prevalence in percentage.

To avoid model overfitting caused by multicollinearity between the selected variables (Dormann et al., 2013), Pearson's correlation analysis was performed and only those variables with correlation coefficient (*r* ^2^) < 0.75 were selected (Appendix S1, Table S1). For instance, if the absolute value of the cross‐correlation coefficient between two variables exceeded 0.75, only the variable that captured more information was selected (Table 1). First, variables that have an *R*_spearman_ less than 0.75 were retained, including bio2, bio15, bio18, and bio19. Actually, if a specific species is studied, among the highly correlated predictors we can retain the variable that has the highest correlation with species occurrence data (Manzoor et al., 2018). Then, we considered the less collinear variables and selected the variable that captured more information. For example, bio14 (precipitation of driest month) and bio17 (precipitation of driest quarter) are highly correlated (*R*_spearma_=0.99, Table S1). Finally, bio17 was selected because of stronger explanation strength than bio14 according to Datta et al. (2019).

**TABLE 1:** Environmental variables used in the MaxEnt model

Code	Description
bio‐2	Mean diurnal range
bio‐10	Mean temperature of warmest quarter
bio‐11	Mean temperature of coldest quarter
bio‐15	Precipitation seasonality
bio‐17	Precipitation of driest quarter
bio‐18	Precipitation of warmest quarter
bio‐19	Precipitation of coldest quarter
BLDFIE_M_sl3_1km_ll	Bulk density (fine earth, oven dry) in kg/cubic‐meter
CECSOL_M_sl3_1km_ll	Cation exchange capacity of soil in cmolc/kg
CLYPPT_M_sl3_1km_ll	Clay content (0–2 micro meter) mass fraction in %
CRFVOL_M_sl3_1km_ll	Coarse fragments volumetric in %
OCDENS_M_sl3_1km_ll	Soil organic carbon density in kg per cubic‐m
ORCDRC_M_sl3_1km_ll	Soil organic carbon content (fine earth fraction) in g per kg
PHIHOX_M_sl3_1km_ll	Soil pH × 10 in H_2_O
PHIKCL_M_sl3_1km_ll	Soil pH × 10 in KCl
SLTPPT_M_sl3_1km_ll	Silt content (2–50 micro meter) mass fraction in %
consensus_full_class_1	Evergreen/deciduous needleleaf trees
consensus_full_class_2	Evergreen broadleaf trees
consensus_full_class_3	Deciduous broadleaf trees
consensus_full_class_4	Mixed/other trees
consensus_full_class_5	Shrubs
consensus_full_class_6	Herbaceous vegetation
consensus_full_class_7	Cultivated and managed vegetation
consensus_full_class_8	Regularly flooded vegetation
consensus_full_class_9	Urban/built‐up
consensus_full_class_10	Snow/ice
consensus_full_class_11	Barren
consensus_full_class_12	Open water
Elevation	–
Slope	–
aspect	–

### 2.2 Species occurrence data

**FIGURE 1:**
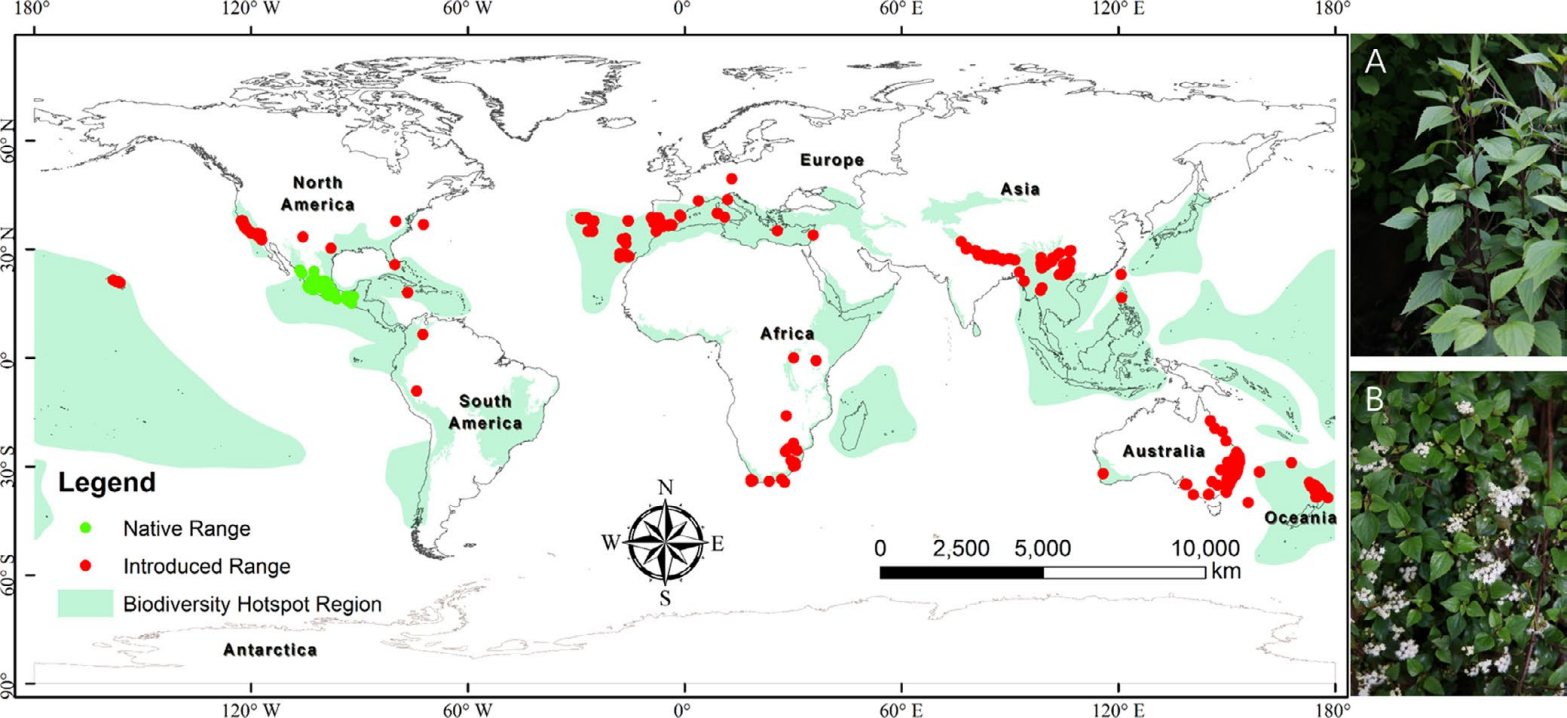
Spatial distribution of *Ageratina adenophora* occurrence. Green points denote native regions, while red points denote introduced or invasive regions. Photographs A and B show *A. adenophora*. The light green‐colored regions show BHRs (updated to the current set of 36 regions: https://www.cepf.net/node/1996; Hrdina & Romportl, 2017), which are Earth's most biologically rich and threatened terrestrial regions (Myers et al., 2000)

Species occurrence data were downloaded from the Global Biodiversity Information Facility (https://www.gbif.org/, accessed 03 September 2018) and the Chinese Virtual Herbarium (http://www.cvh.ac.cn/, accessed 03 September 2018). Furthermore, we collected 10 samples from Gyirong and Nyalam counties, which are adjacent to Nepal, during the fieldwork in 2016. A total of 5,474 occurrence points were initially recorded. Occurrence records are often biased toward geographically convenient or environmentally friendly (e.g., areas near cities or areas with high population density), resulting in sampling bias in geographic space. Thus, spatial thinning was performed to remove the spatial autocorrelation and sampling bias. Grid cells with dimensions of 10 × 10 km were created, and a single occurrence point was selected randomly from each cell with more than one occurrence point (Ahmad et al., 2019). A total of 741 unbiased occurrence data points from regions in Asia (74 points), Africa (68 points), Australia (344 points), Oceania (70 points), North America (101 points in total and 48 points from native ranges), and South America (two points) were saved in CSV format (Figure 1). The native and introduced regions were delineated according to the biogeographical distribution scheme of the United States Department of Agriculture's Germplasm Resource Information Network (https://npgsweb.ars‐grin.gov, accessed 04 September 2019).

### 2.3 Modeling approach and spatial analysis

We applied Maxent, version 3.3.3k (available at http://biodiversityinformatics.amnh.org/open_source/maxent/; Phillips et al., 2006) to predict the potential suitable area of *A*. *adenophora*. As one of the most effective presence‐only algorithms available, Maxent has been shown to perform better than other models, and it is quite robust when there are a small number of occurrence points (Elith et al., 2006; Hu et al., 2015; Jarnevich et al., 2010; Wisz et al., 2008; Yu, Zhang, et al., 2014). Seventy percent of the occurrence points were selected for model training, while the other 30% were used for model validation. The model output represented the probability of presence from 0 to 1 (Phillips & Dudík, 2008). The area under the curve (AUC) of the receiver operating characteristic (ROC) curve was used to evaluate the model performance. The AUC value ranges from 0 to 1, an AUC value between 0.5 and 0.7 indicates that the model performance is not acceptable, AUC in the range of 0.7–0.9 indicates good performance, and AUC > 0.9 indicates the highest predictive ability (Abdelaal et al., 2019; Phillips et al., 2006). Furthermore, we also calculated the true skill statistics (TSS) to estimate the model performance (Allouche et al., 2006; Fielding & Bell, 1997; Swets, 1988). As a threshold‐dependent metric of model evaluation, TSS ranges from −1 to +1 and values above 0.75 indicate excellent model performance (Allouche et al., 2006).

The most commonly used framework combines occurrence records from both the native and introduced regions by using distribution data from the native range, this strategy makes use of those occurrence records that are likely to be in equilibrium with the regional environment while also including records from introduced regions which may provide additional information about expansion into novel ranges (Marcelino & Verbruggen, 2015; Wan et al., 2017). Four arbitrary categories of invasion risk for *A*. *adenophora* were defined as no risk (NR, <0.2), low risk (LR, 0.2–0.4), moderate risk (MR, 0.4–0.6), and high risk (HR, >0.6) based on predicted habitat suitability (Xu et al., 2019; Zhang et al., 2018, 2019). In this study, we defined a region as an under‐risk (UR) region when its risk category was LR, MR, or HR. Furthermore, *Ageratina adenophora* is native to Mexico; therefore, occurrence of this species in Mexico is not due to invasion. Thus, we masked out Mexico when calculating the UR regions. Based on the predicted results for the current climate conditions and eight RCPs, the risks of invasion by *A. adenophora* in different areas were calculated using ArcGIS 10.4.1 based on the four arbitrary categories defined above. To explore the variation in the distribution of *A. adenophora* with altitude under climate change scenarios, we calculated the areas and area ratio of LR, MR, HR, and UR in different elevation ranges under climate change scenarios and applied the “ecospat” package in R to visualize these changes (Di Cola et al., 2017).

## 3 RESULT

### 3.1 Model performance and main variables

**FIGURE 2:**
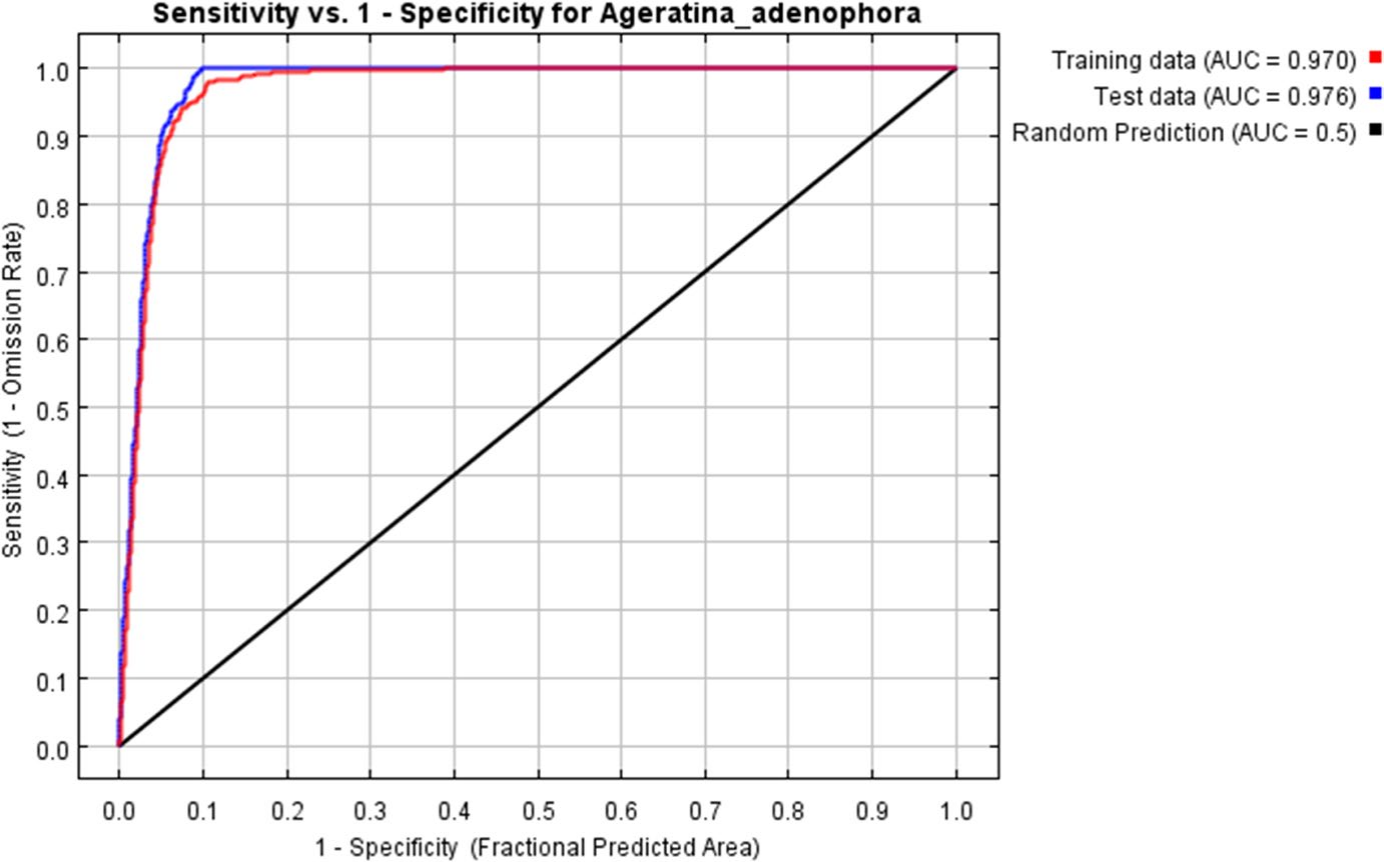
ROC curve and AUC value under current climate conditions

The AUC value for *A. adenophora* obtained using the MaxEnt model was 0.97 (Figure 2), indicating excellent model performance. The TSS value was 0.82, further supporting the reliability of the results. The jackknife test of the model indicated that the following major variables contribute significantly to the potential suitability of *A*. *adenophora* (Table 2): mean temperature of coldest quarter (47.5%), evergreen broadleaf trees (22.9%), urban/built‐up (6.5%), barren (5.8%), mean temperature of warmest quarter (2.8%), cation exchange capacity of soil (2.2%), soil pH (1.4%), coarse fragments volumetric (1.3%), and precipitation seasonality (1.1%). Among the variable types, climate factors made the largest contribution to the potential suitability of *A. adenophora* in our model (51.4%), with mean temperature of coldest quarter having the largest contribution (47.5%). Land‐cover variables were the second most influential, with evergreen broadleaf trees having the greatest contribution among land‐cover factors. Soil conditions and terrain factors had relatively small contributions to the potential suitability of *A. adenophora*.

**TABLE 2:** Main variables in the MaxEnt model of *Ageratina adenophora* under current climate conditions

Variable	Percent contribution	Permutation importance
Mean temperature of coldest quarter (bio_11)	47.5	47.5
Evergreen broadleaf trees (consensus_full_class_2)	22.9	70.4
Urban/built‐up (consensus_full_class_9)	6.5	76.9
Barren (consensus_full_class_11)	5.8	82.7
Mean temperature of warmest quarter (bio_10)	2.8	85.5
Cation exchange capacity of soil in cmolc/kg (cecsol_m_sl3_1km_ll)	2.2	87.7
Soil pH × 10 in H_2_O (phihox_m_sl3_1km_ll)	1.4	89.1
Coarse fragments volumetric in % (crfvol_m_sl3_1km_ll)	1.3	90.4
Precipitation seasonality (bio_15)	1.1	91.5

**FIGURE 3:**
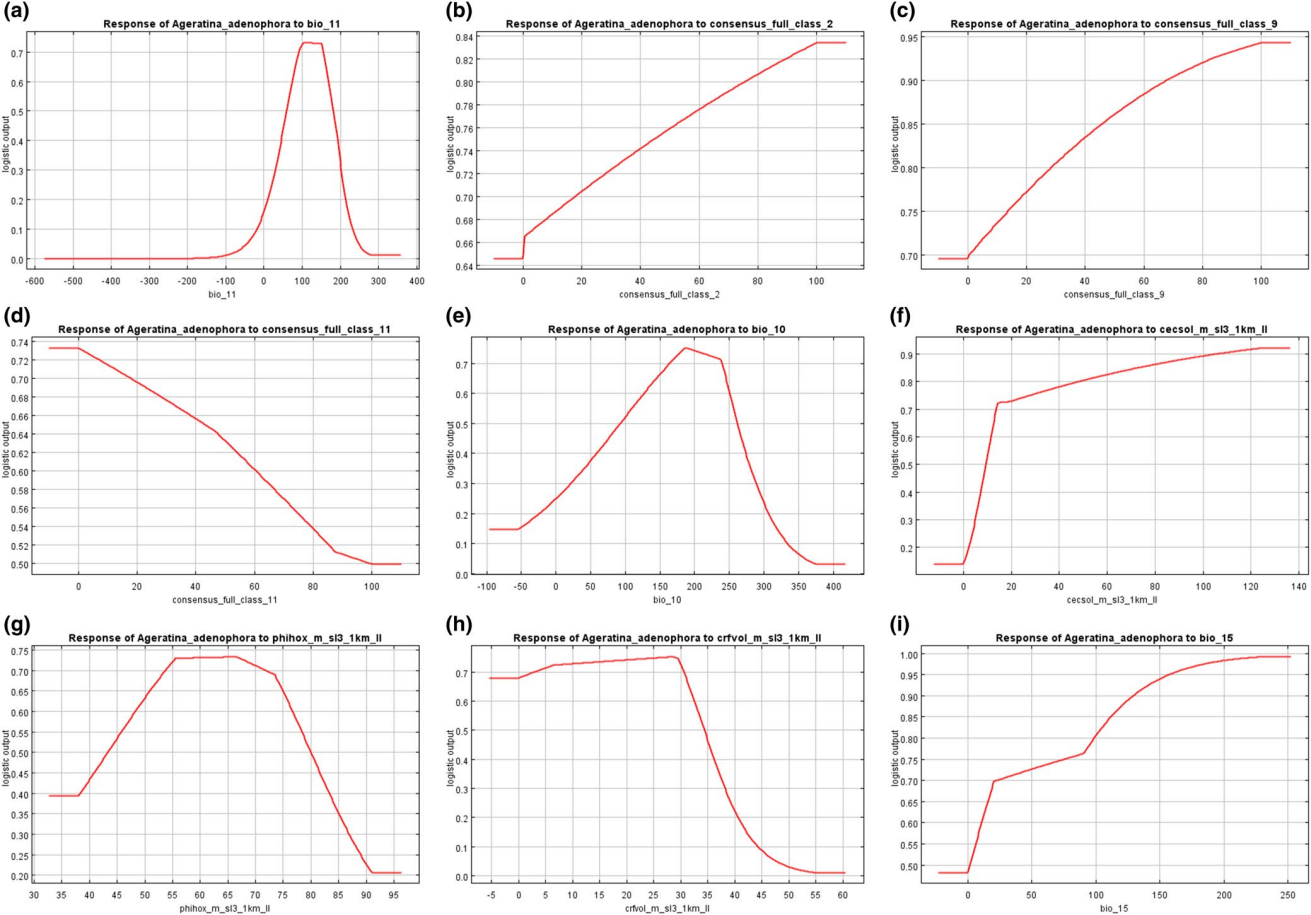
Response curves for the eight main environmental variables affecting the potential suitable area of *Ageratina adenophora*. The thresholds of suitability were set as existence probability greater than 0.2

Based on the response curves of the eight environmental variables to potential suitability (Figure 3), the potential suitable ranges with respect to the different variables were observed. The suitable mean temperature of coldest quarter ranges from 2°C to 22°C. From the view of land‐cover types, *A. Adenophora* is adapted to evergreen broadleaf trees and urban/built‐up regions. The potential suitability of *A. Adenophora* increases with increasing evergreen broadleaf trees and urban/built‐up but decreases with decreasing barren land. The suitable range of mean temperature of warmest quarter is −2.5°C to 31°C. The probability of *A. Adenophora* potential suitability increases with increasing soil Cation Exchange Capacity. The optimal soil pH for *A. adenophora* ranges from 4.8 to 7.7, with maximum germination occurring at pH about 6.7. As soil coarse fragments volumetric increases, the potential suitability of *A. adenophora* decreases, particularly for values exceeding 20%. Precipitation seasonality (coefficient of variation) has little influence on the distribution of *A*. *adenophora*. According to the model, temperature had a strong effect on the potential suitability of *A. Adenophora*, and this species prefers a warm climate. Compared with temperature and land‐cover variables, soil conditions and precipitation factors have little effect on the potential suitability of *A. adenophora*.

### 3.2 Current invasion pattern of *A*. *adenophora*

**FIGURE 4:**
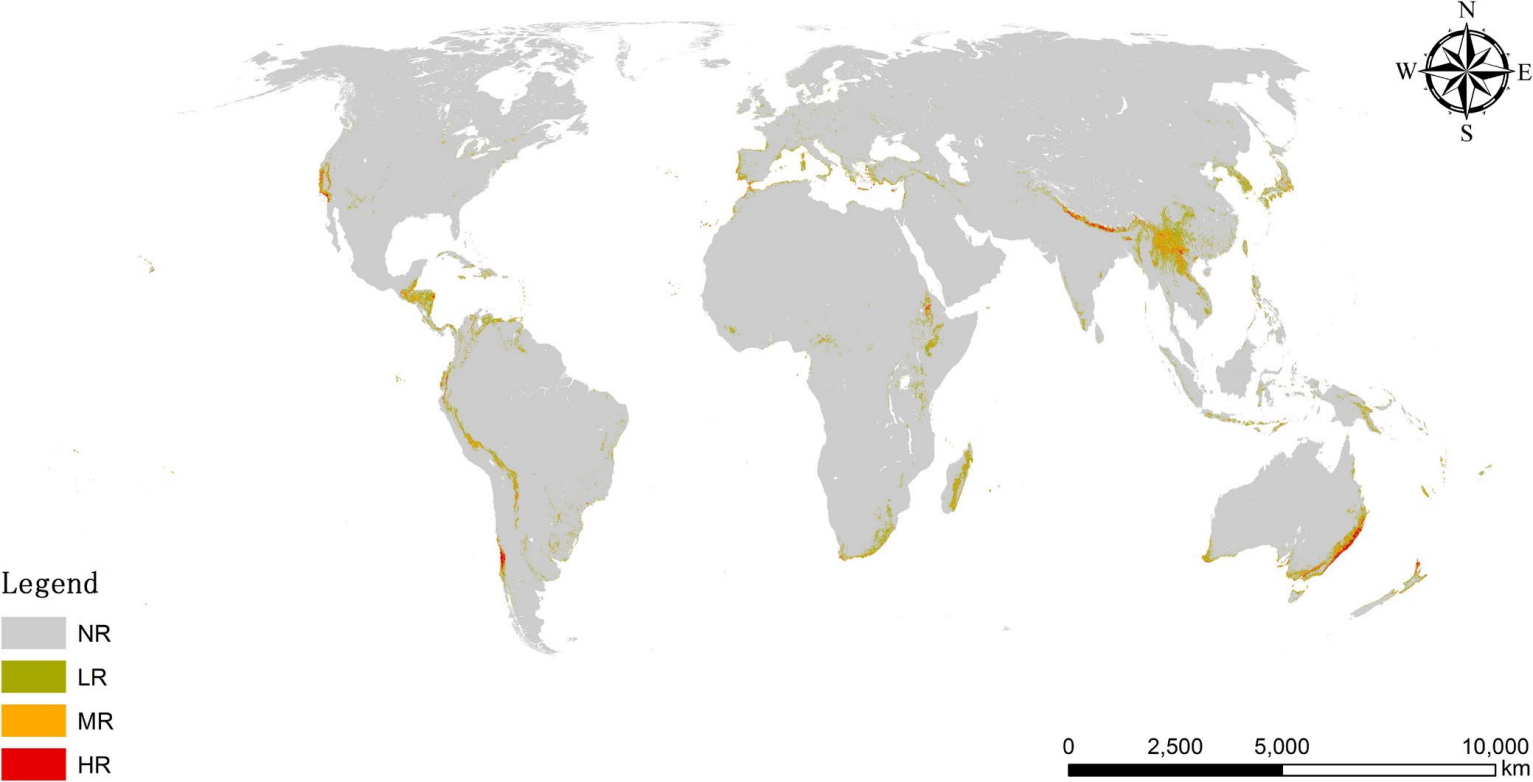
Potential suitable area of *Ageratina adenophora* under current climate conditions. NR, LR, MR, and HR denotes no risk, low risk, moderate risk, and high risk, respectively

Figure 4 shows the percentages of areas in different risk categories under current climate conditions. According to the global map of potential suitable areas of *A. Adenophora*, the total area of UR regions was 4,785,146 km^2^, of which 2,638,156 km^2^ was classified as HR. The areas of MR and LR regions were 1,076,438 km^2^ and 3,444,893 km^2^, respectively. Most UR regions for *A. adenophora* invasion were located in the western coastal part of the United States, the southern part of Chile, the central parts of Peru and Bolivia, the southern coastal part of South Africa, most parts of Ethiopia and Madagascar, the eastern coastal part of Australia, most parts of the central Himalaya in India and Nepal, the southwestern region of China, most of Taiwan, the eastern parts of Myanmar, most parts of Laos and the Korean peninsula, and large parts of Japan. Among these regions, the regions classified as HR are mainly distributed in Chile, the eastern coastal part of Australia, and the central Himalayas.

**FIGURE 5:**
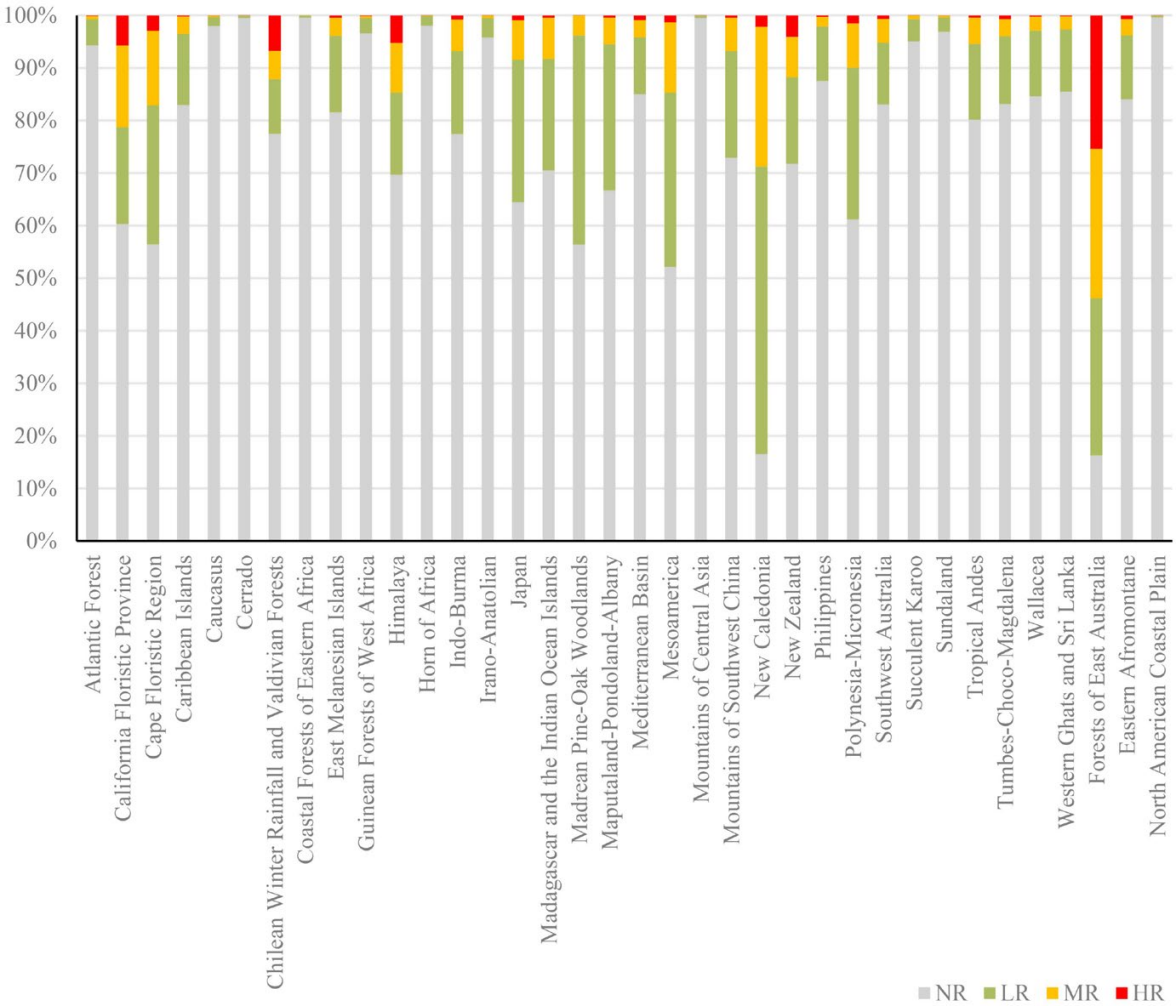
Potential invasion areas within BHRs under current climate conditions. NR, LR, MR, and HR denotes no risk, low risk, moderate risk, and high risk, respectively

There are currently 36 recognized biodiversity hotspot regions (BHRs) worldwide. Figure 5 shows the estimated potential invasion range of *A. adenophora* in these BHRs. At present, 3,298,008 km^2^ of the UR area is found in BHRs, accounting for approximately 69% of the total UR area in the world; the BHR areas classified as LR, MR, and HR are 2,251,115, 813,817, and 233,076 km^2^, respectively (Table 3). The BHR areas classified as LR, MR, and HR account for approximately 65.35%, 75.60%, and 88.35% of the total worldwide areas classified as LR, MR, and HR, respectively. The BHR containing the largest UR area (531,980 km^2^) is the Indo‐Burma BHR in South‐East Asia, which comprises all nonmarine parts of Cambodia, Laos, Myanmar, Thailand, and Vietnam along with parts of southern China. This area also contains the largest LR area (372,109 km^2^) and MR (141,324 km^2^) area and is one of the most biologically important regions on the planet. Among BHRs, the Forests of East Australia BHR has the largest area is classified as HR (64,042 km^2^) and also the largest UR proportion (83.72%) of the total area. This BHR consists of a discontinuous coastal stretch along the Australian states of Queensland and New South Wales and extends inland and further west to include the New England Tablelands and the Great Dividing Range. The areas classified as HR and MR in the Forests of East Australia BHR account for approximately 26% and 29% of the BHR’s total area, respectively. The BHR containing the smallest UR area (1,263 km^2^) is the Coastal Forests of Eastern Africa, which stretches along the eastern edge of Africa and includes parts of Somalia, Kenya, Tanzania, and Mozambique. This area also contains the smallest LR area (1,249 km^2^), MR (14 km^2^) area, and zero HR area.

**TABLE 3:** Area in square kilometers (km^2^) in the BHR areas classified as different risk rank

Code	Name	NR (km^2^)	LR (km^2^)	MR (km^2^)	HR (km^2^)	UR (km^2^)
1	Atlantic Forest	1,152,535	60,733	7,571	1,553	69,856
2	California Floristic Province	169,465	51,633	43,824	16,060	111,518
3	Cape Floristic Region	44,174	20,672	11,100	2,293	34,065
4	Caribbean Islands	184,666	30,109	7,434	469	38,012
5	Caucasus	519,531	8,915	1,497	272	10,684
6	Cerrado	2,011,961	9,663	611	34	10,307
7	Chilean Winter Rainfall and Valdivian Forests	301,945	40,359	21,164	26,199	87,722
8	Coastal Forests of Eastern Africa	287,167	1,249	14	0	1,263
9	East Melanesian Islands	77,929	13,913	3,328	415	17,656
10	Guinean Forests of West Africa	595,023	17,695	2,705	579	20,979
11	Himalaya	489,142	109,690	66,367	36,661	212,719
12	Horn of Africa	1,623,034	29,866	2,374	33	32,272
13	Indo‐Burma	1,821,543	372,109	141,324	18,547	531,980
14	Irano‐Anatolian	849,752	32,582	4,871	79	37,532
15	Japan	238,385	100,252	27,732	3,508	131,492
16	Madagascar and the Indian Ocean Islands	420,442	126,506	46,880	2,634	176,020
17	Madrean Pine–Oak Woodlands	6,388	4,499	434	1	4,934
18	Maputaland–Pondoland–Albany	180,870	75,379	13,810	1,093	90,283
19	Mediterranean Basin	1,763,748	223,846	68,646	18,604	311,096
20	Mesoamerica	259,306	164,617	66,400	6,685	237,703
21	Mountains of Central Asia	816,558	3,630	444	11	4,086
22	Mountains of Southwest China	190,310	52,965	16,599	1,158	70,721
23	New Caledonia	3,042	10,066	4,897	397	15,360
24	New Zealand	191,442	43,917	20,595	10,892	75,405
25	Philippines	256,567	30,418	5,333	854	36,605
26	Polynesia‐Micronesia	25,765	12,165	3,556	638	16,359
27	Southwest Australia	294,152	41,622	15,989	2,446	60,057
28	Succulent Karoo	97,280	4,320	705	24	5,050
29	Sundaland	1,439,982	39,722	5,751	497	45,969
30	Tropical Andes	1,221,793	219,068	76,561	6,411	302,040
31	Tumbes–Choco–Magdalena	224,835	34,726	8,871	1,923	45,521
32	Wallacea	280,521	41,337	8,887	754	50,978
33	Western Ghats and Sri Lanka	160,486	22,260	4,677	343	27,279
34	Forests of East Australia	41,048	75,403	71,642	64,042	211,088
35	Eastern Afromontane	837,605	121,486	30,904	6,964	159,353
36	North American Coastal Plain	1,108,392	3,726	318	1	4,045
Total		20,186,786	2,251,115	813,817	233,076	3,298,008

### 3.3 Potential suitable area of *A. adenophora* under different future climate change scenarios

**FIGURE 6:**
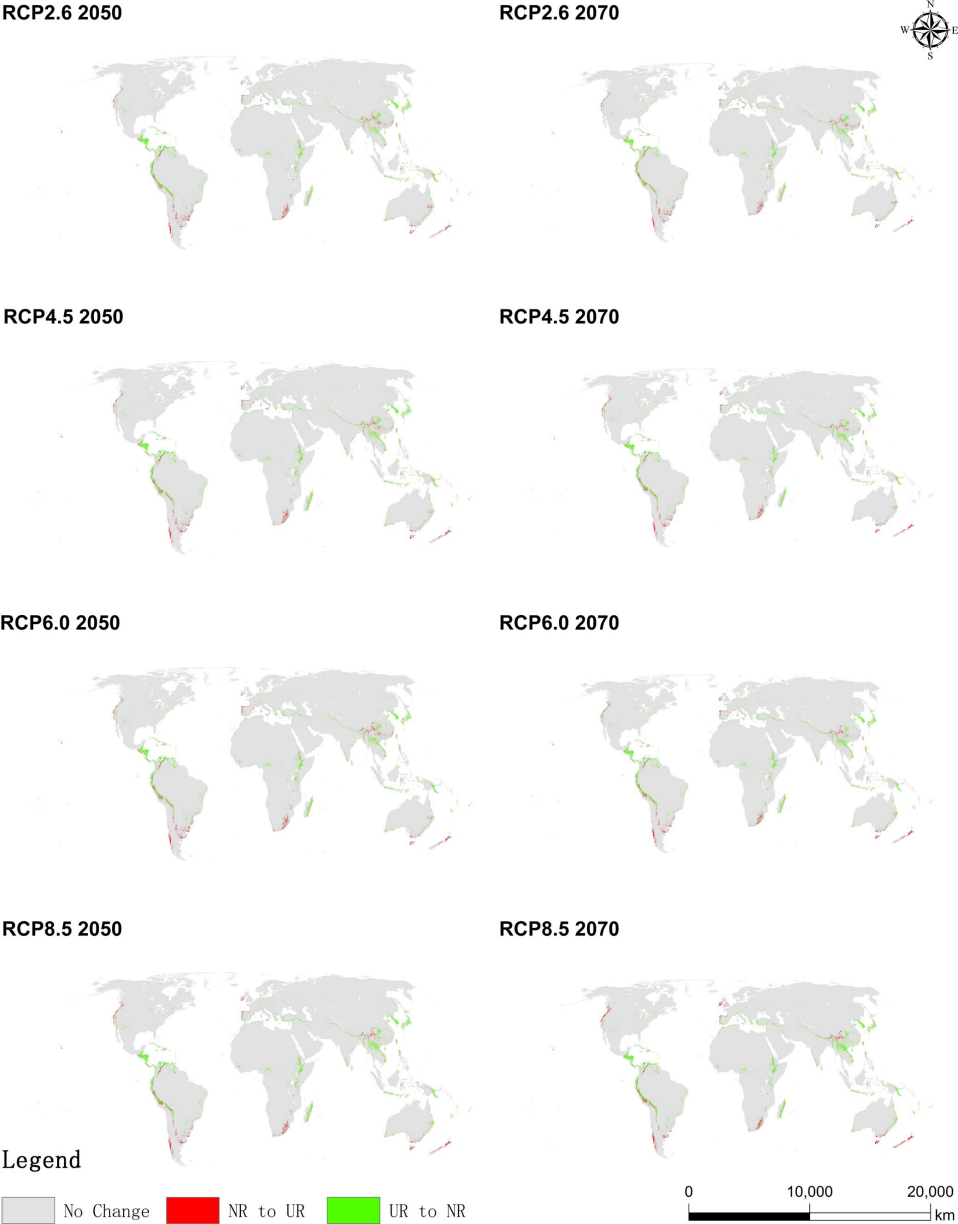
Potential suitable area of *Ageratina adenophora* under the eight RCPs. Gray denotes no risk, and green denotes regions converted from UR to NR; red denotes regions converted from NR to UR

The potential suitable regions for *A. adenophora* invasion were analyzed under the eight different future climatic scenarios (RCP2.6, RCP4.5, RCP6.0, and RCP8.5 in 2050 and 2070). The results indicate that the potential suitable area of *A. adenophora* will shrink under all RCPs (Figure 6, Table 4). Compared with current conditions, the increase in the area classified as NR ranged from 1.14% under RCP2.6 2070 to 1.54% under RCP8.5 2070. The area classified as LR decreased under all RCPs compared with current conditions, with the decrease ranging from 31.18% (RCP2.6 2070) to 45.58% (RCP8.5 2070) with an average of 36.32% (the largest decline among risk categories). The areas classified as MR and HR also decreased with respect to current conditions, with average decreases of 24.86% and 22.22%, respectively. Analyzing the spatial patterns of the potential suitable regions indicated that the decreases in areas classified as LR and MR compared with current conditions were mainly distributed in Guatemala, Nicaragua, Costa Rica, the central parts of Peru and Bolivia, the southern parts of Chile, Nigeria, the southern parts of South Africa, western Madagascar, the central Himalaya, western and eastern Myanmar, Northern Laos, southwestern China, the entire Korean Peninsula, and Japan (Figure 6). Although the UR areas in different categories generally showed the same shrinking trend under the future climate change scenarios, the opposite trend was observed in some regions. For example, some areas categorized as NR or LR for *A. adenophora* invasion under current climate conditions will become MR or even HR areas under future climate change scenarios, including northwestern California, southern Chile, southern South Africa, the central Himalaya, and southwestern China. In summary, the regions suitable for *A. Adenophora* invasion (those classified as LR, MR, or HR) decreased under the future climate change scenarios, including Guatemala, Nicaragua, Costa Rica, the central parts of Peru and Bolivia, the southern parts of Chile, Nigeria, the southern parts of South Africa, western Madagascar, the central Himalaya, western and eastern Myanmar, Northern Laos, southwestern China, the entire Korean Peninsula, and Japan, although the opposite trend was observed on a regional scale.

**TABLE 4:** Area in square kilometers (km^2^) and rate of changes in the areas classified as different risk rank under future climatic scenarios for two time periods (2050 and 2070)

Risk rank	Current (km^2^)	RCP2.6 (%)		RCP4.5 (%)		RCP6.0 (%)		RCP8.5 (%)	
		2050	2070	2050	2070	2050	2070	2050	2070
No risk (NR)	123,766,200	1.16	1.14	1.24	1.28	1.17	1.29	1.38	1.54
Low risk (LR)	3,444,893	−31.52	−31.18	−34.76	−36.07	−33.61	−38.19	−39.62	−45.58
Moderate risk (MR)	1,076,438	−25.20	−24.84	−25.37	−25.63	−22.65	−22.83	−26.07	−26.32
High risk (HR)	263,815	−27.83	−26.36	−22.34	−25.61	−18.33	−12.51	−24.52	−20.23
Under risk (UR)	4,785,146	−29.90	−29.49	−31.96	−33.15	−30.30	−33.32	−35.74	−39.85

**FIGURE 7:**
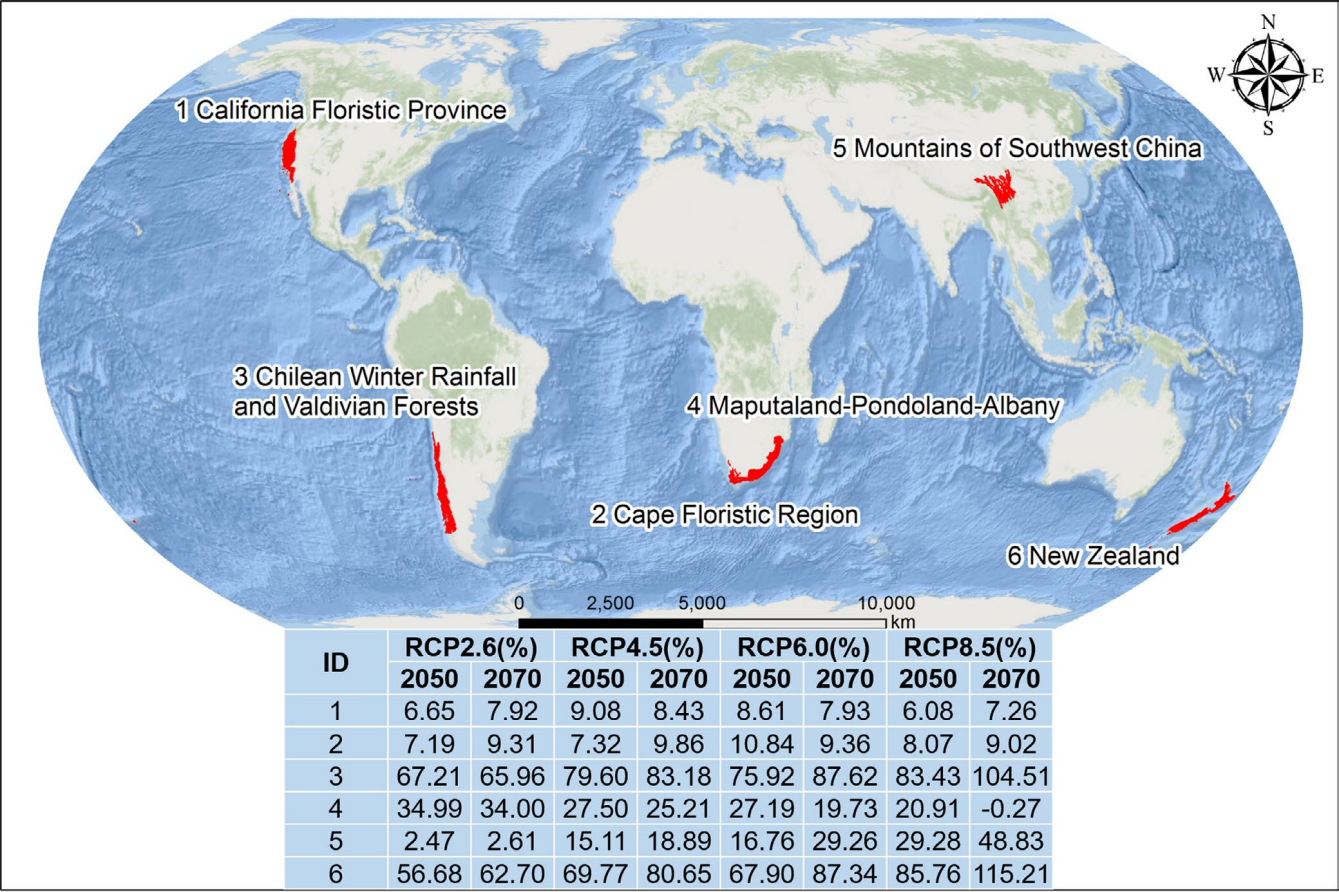
Changes in UR area in six BHRs under the eight RCPs. 1, California Floristic Province Cape Floristic Region; 2, Chilean Winter Rainfall and Valdivian Forests; 3, Maputaland–Pondoland–Albany; 4, Mountains of Southwest China; 5, New Zealand; 6, North American Coastal Plain

Similarly, the UR area within BHRs decreased under the future climate change scenarios compared with under current conditions. According to the predicted results, the UR area in BHRs will decrease from 3,298,008 km^2^ under current conditions to an average of 2,118,453 km^2^ under the eight RCPs, with the largest decrease (decreased by 1,467,862 km^2^) occurring under RCP8.5 in 2070. The average decreases in areas classified as LR, MR, and HR within BHRs were 38.66%, 30.87%, and 24.94%, respectively, with the largest corresponding decreases being 49.65% under RCP8.5 in 2070, 35.06% under RCP8.5 in 2070, and 28.07% under RCP8.5 in 2050, respectively. In 29 out of 36 BHRs, the UR area decreased under the future climate change scenarios with respect to under current conditions, and the largest average decrease occurred in the Indo‐Burma BHR (220,881 km^2^). Increases in UR area were observed in only six BHRs: California Floristic Province, Cape Floristic Region, Chilean Winter Rainfall and Valdivian Forests, Maputaland–Pondoland–Albany, Mountains of Southwest China, and New Zealand. As shown in Figure 7, obvious increasing trends in UR area can be observed in the Chilean Winter Rainfall and Valdivian Forests, New Zealand, and Mountains of Southwest China BHRs; the UR areas in the California Floristic Province and Cape Floristic Region BHRs remained relatively flat. Among the BHRs, the largest increase (increased by 115.21%) in the UR area was found in the Maputaland–Pondoland–Albany BHR under RCP8.5 in 2070.

### 3.4 *A. Adenophora* potential suitable area characteristics with an elevation under current conditions and climate change scenarios

Under current conditions, the UR regions are mainly distributed at elevations below 2,500 m; these regions account for approximately 97% of the total UR area, with areas at elevations under 500m accounting for 38.82% (Table 5). The areas classified as LR, MR, and HR show similar distributions with elevation; these areas are primarily distributed in low‐elevation regions, and the potential suitability of *A. adenophora* decreases with increasing elevation. Regions with elevations below 2,500 m are also the main potential suitable areas of *A. Adenophora* under the eight RCPs. UR areas at elevations below 2,500 m decreased under all RCPs compared with under current conditions. The UR areas at elevations between 500 and 1,000 m are currently around 1,127,147 km^2^; this value decreased by an average of 53.15% under the eight RCPs, with the largest decrease (64.13%) occurring under RCP8.5 in 2070. The URs at elevations between 1,000 and 1,500 m decreased by an average of 41.47% under the eight RCPs. The URs at elevations between 1,500 and 2,000 m decreased by an average of 24.16% under all RCPs compared with under current conditions. This decreasing trend is getting small with the elevation rise up. For example, UR areas at elevations between 2,000 and 2,500 m decreased by only an average of 8.08% under eight RCPs. However, increasing trend was observed in UR areas above 2,500 m. The UR areas with elevations between 2,500 and 3,000 m increased by an average of 43.84% under the eight RCPs. This value increased to 142.16% when UR areas with elevations between 3,000 and 3,500 m. When the UR areas are at an altitude of more than 3,500 m but lower than 4,000 m, UR areas increased by an average of 363% under the eight RCPs, it only takes no more than 0.2% of the total UR areas. The UR areas with elevations higher than 4,000 m increased by an average of 214.66% under RCP4.5, RCP6.0, and RCP8.5 but decreased by an average of 49.99% under RCP2.6.

**TABLE 5:** Distributions of UR regions in different elevation ranges under current conditions and the eight RCPs

Elevation (m)	Current (km^2^)	RCP2.6 2050 (%)	RCP2.6 2070 (%)	RCP4.5 2050 (%)	RCP4.5 2070 (%)	RCP6.0 2050 (%)	RCP6.0 2070 (%)	RCP8.5 2050 (%)	RCP8.5 2070 (%)
<500	1,857,734	−29.05	−28.26	−31.54	−31.67	−30.45	−33.72	−35.50	−38.66
500–1,000	1,127,147	−47.19	−47.00	−51.82	−53.97	−49.53	−54.23	−57.34	−64.13
1,000–1,500	865,412	−35.34	−35.35	−38.75	−41.74	−36.57	−41.68	−45.16	−57.16
1,500–2,000	525,626	−23.23	−22.30	−23.56	−24.98	−22.02	−23.65	−25.38	−28.16
2,000–2,500	259,966	−5.86	−5.37	−6.63	−9.55	−4.25	−7.54	−9.92	−15.55
2,500–3,000	104,114	34.76	32.89	43.73	43.43	45.32	50.15	47.90	52.50
3,000–3,500	34,051	94.38	90.91	131.68	140.39	130.17	161.56	160.51	227.70
3,500–4,000	8,971	188.77	185.32	292.64	366.96	285.55	419.79	424.02	740.95
>4,000	2,126	−49.64	−50.34	15.36	94.35	3.88	135.88	151.33	887.14

The same phenomenon was observed in the areas classified as LR, MR, and HR (Appendix S1, Tables S2–S4). Under current climate conditions, the areas classified as LR, MR, and HR are primarily distributed in regions with elevations below 2,500 m (96.63%, 97.31%, and 98.47%, respectively) and areas at elevations under 500m accounting for 38.82%, 34.64%, and 55.95%, respectively. Under all RCPs, LR areas with elevations below 2,500 m, MR areas with elevations below 2,000 m, and HR areas with elevations below 1,500 decreased compared with under current conditions. The greatest average decreases of areas under different risk ranks were found in different altitude intervals. As for HR and MR areas, the greatest average decreases were found in regions between 1,000 and 1,500 m. The LR areas with elevations between 500 and 1,000 m decreased most, by an average of 50.79%, under the eight RCPs. Oppose trend was observed in mid‐high elevation regions: For example, LR areas increased when LR areas with elevations between 2,500 and 3,500 m and the greatest increases were found between 3,500 and 4,000 m (an average of 360.91% increase). As elevation increases (higher than 4,000 m), LR areas increased by an average of 235.91% except under RCP2.6. Similar to LR areas, MR areas increased when MR areas with elevations between 2,000 and 4,000 m and the greatest increases were found between 3,500 and 4,000 m (an average of 468.38% increase). MR areas with elevations higher 4,000 m, increase trend can only be observed under RCP6.0 in 2070 and RCP8.5. Unlike the above two risk ranks, HR areas with elevations between 1,500 and 2,000 m increased under RCP4.5 in 2050 and RCP6.0. As elevation increases (between 2,000 and 2,500 m), HR areas increased under RCP4.5 in 2050, RCP6.0, and RCP8.5. When the HR areas are at an altitude of more than 2,500 m but lower than 3,000 m, HR is increased under all RCPs except for RCP2.6 in 2050. The greatest increases were found in regions with elevations between 3,000 and 3,500 m, increased by an average of 132.11%. According to the above results, we found that regions with elevations between 3,000 and 3,500 experienced the greatest growth of all risk ranks.

### 3.5 Dynamics in potential suitable area of *A. Adenophora* under climate change scenarios

**FIGURE 8:**
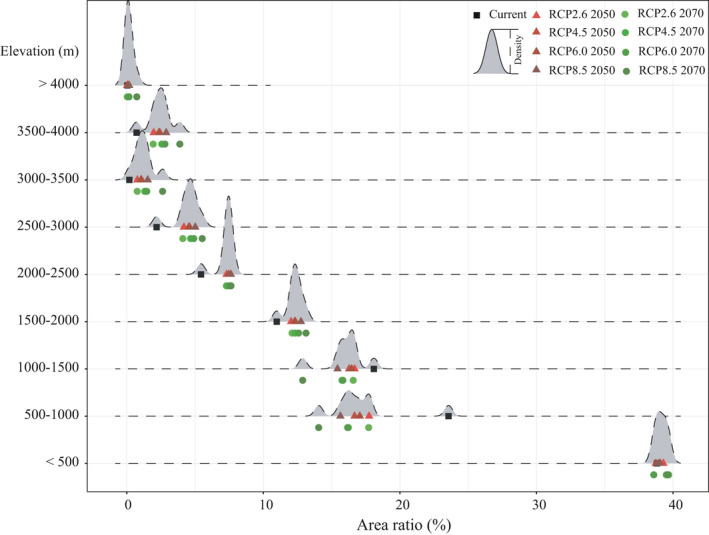
Distributions of UR regions for *Ageratina adenophora* within different elevation ranges. Red triangles denote the four RCPs in 2050, while the green dots represent the four RCPs in 2070. To improve the visibility of differences between the RCPs for 2050 and 2070, the four RCPs for 2070 are located beneath the four RCPs for 2050

To analyze the potential suitable area shifts of *A. Adenophora,* a further analysis about the potential suitable areas of *A. Adenophora* along with the elevation is depicted in Figure 8. Compared with under current conditions, the percentages of UR areas at elevations between 500 and 1,500 m clearly decreased under future climate scenarios, while the opposite trend was observed for UR areas at elevations above 1,500 m or below an elevation of 500 m except for RCP6.0. The percentages of UR areas at elevations between 500 and 1,000 m decreased at an average of 7.15% under future climate change scenarios; this number is 2.35% in areas at elevations between 1,000 and 1,500 m and 2.35% in areas at elevations between 1,000 and 1,500 m. When the altitude is higher than 1,500 m, percentages of UR areas increased and the greatest average increase was found in elevation ranges between 2,500 and 3,000 m. The same trend was also observed in MR and HR areas (Appendix S2, Figure S1 and Table S2). The percentages of MR and HR areas at elevations between 500 and 1,500 m clearly decreased. The greatest decrease was found in elevation ranges between 500 and 1,000 m, with an average decrease of 10.82% and 5.97%, respectively. Percentages of MR areas increased when areas with elevations above 1,500 m and the greatest increase were observed in elevations ranges between 2,000 and 2,500 m. Percentages of HR areas increased when areas with elevations between 1,500 and 4,000 m except areas with elevations between 1,500 and 4,000 m of RCP8.5 in 2070. Percentages of LR areas decreased when areas at elevations below 1,500 m and the greatest decrease were found in elevations ranges between 500 and 1,000 m (Appendix S2, Figure S3). When the altitude is higher than 1,500 m but lower than 4,000 m, percentage of LR increased compared with current conditions.

**FIGURE 9:**
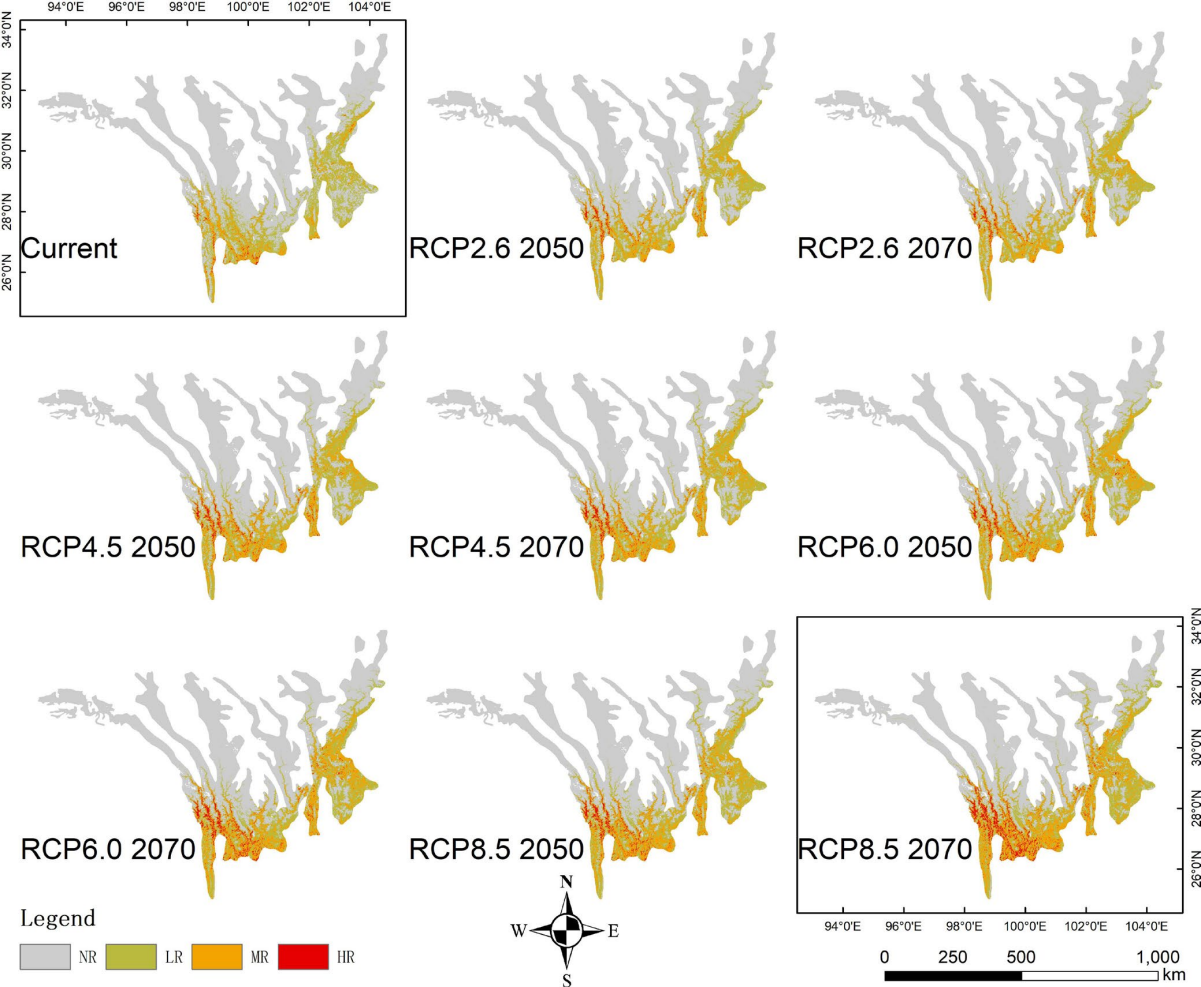
Potential invasion range of *Ageratina adenophora* in the Mountains of Southwest China BHR under current and future climate change scenarios. The trend in *A. adenophora* invasion range in this BHR is opposite the global trend

**FIGURE 10:**
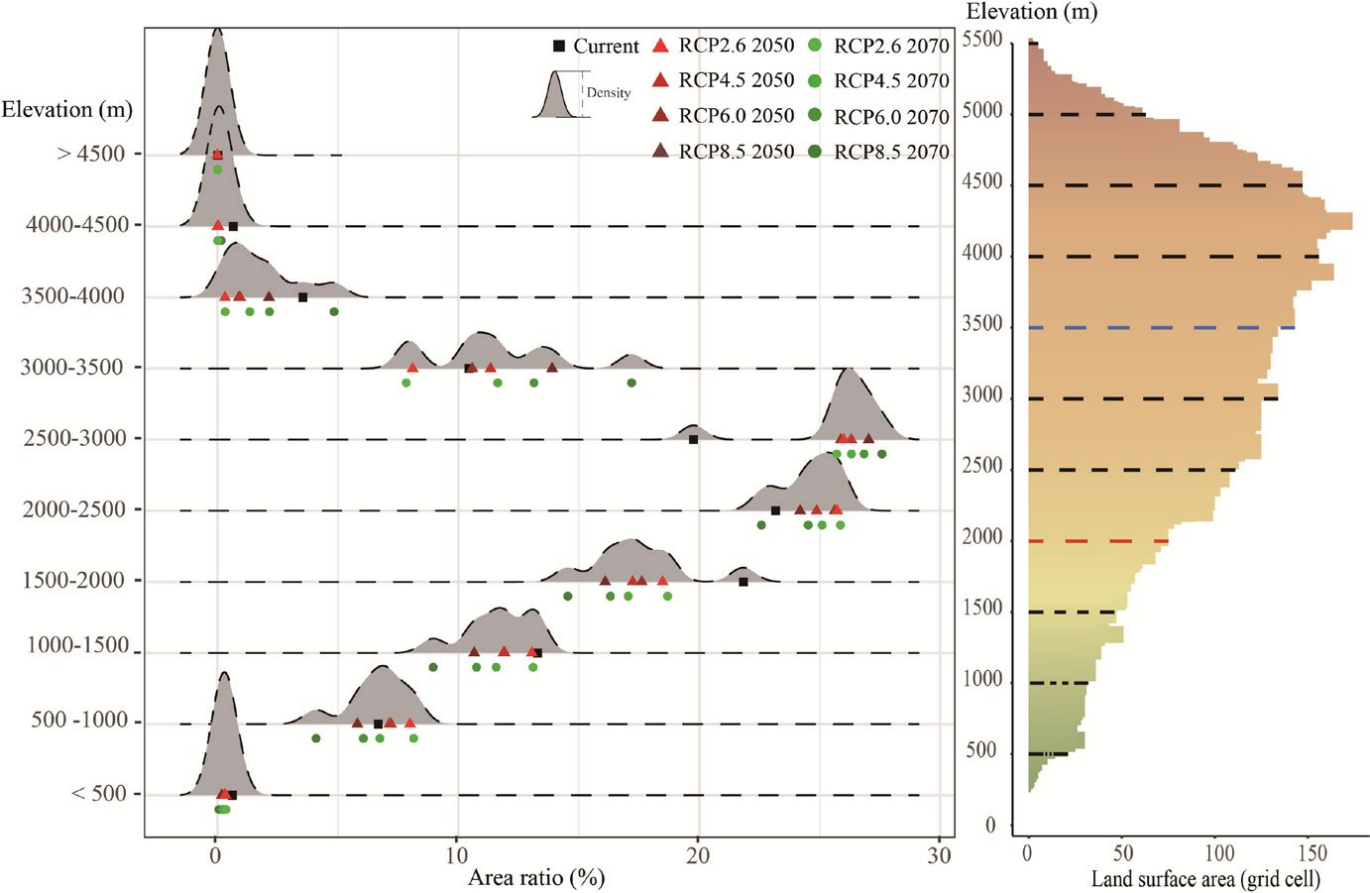
Distributions of *Ageratina adenophora* UR regions in different elevation ranges within the Mountains of Southwest China BHR

**FIGURE 11:**
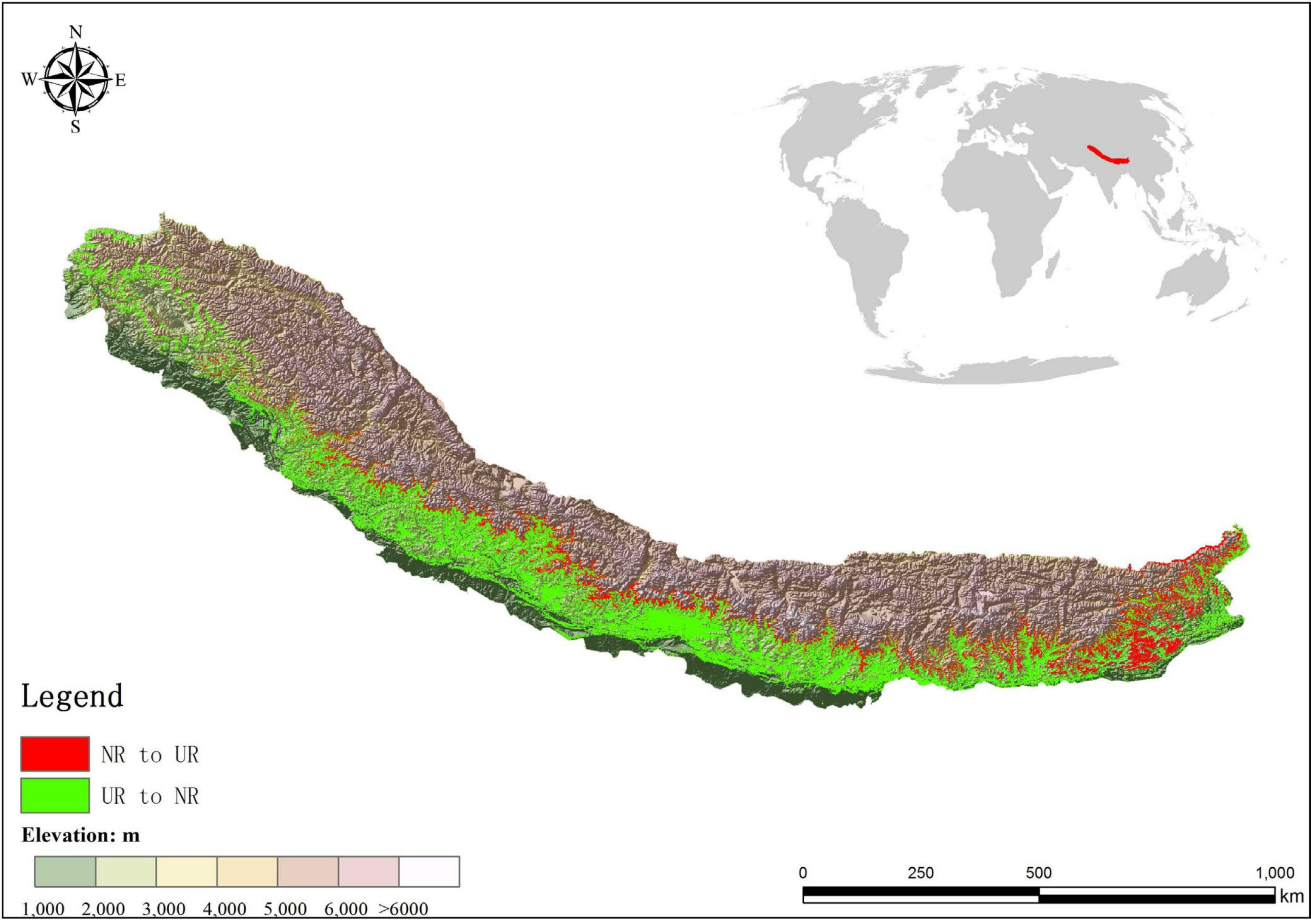
Distributions of *Ageratina adenophora* UR regions in the Himalayas under current conditions and a future climate change scenario (RCP8.5 2070)

Furthermore, we found similar potential suitable areas shift of *A. adenophora* under the future climate change scenarios in the regional scale. Mountains of Southwest China BHR (Figures 9 and 10), which has suffered severe damage due to the invasion of *A. adenophora*, with areas at elevations of 2,500–3,000 m accounting for the largest proportion of UR areas (average of 26.46%) under all RCPs except RCP 2.6 2070. It is worth noting that the UR areas at elevations below 2,000 m decreased under all RCPs compared with under current conditions. For example, UR areas with elevations below 2,000 m account for 42.41% of all UR regions under current conditions; this percentage decreased to 27.65% under RCP8.5 2070. Nevertheless, UR areas with elevations between 2,000 and 3,500 m increased under all RCPs. Under current climate conditions, the UR areas are primarily distributed at elevations of 2000–2500. However, under RCP8.5 2070, the UR areas are primarily found at elevations of 2,500–3,000 m. This phenomenon was also observed in the Himalayas (Figure 11). As shown in Figure 11, *A. adenophora* shows an obvious trend of expansion at higher altitudes in the Himalayan region. For example, under RCP 8.5 in 2070, the percentage of UR areas at elevations between 3,000 and 3,500 m (3.58%) is nearly fivefold higher than that under current climate conditions.

## 4 DISCUSSION

IAS has caused enormous economic losses and threatens biodiversity globally. The continental accumulation of IAS is predicted to increase by 36% from 2005 to 2050 (Seebens et al., 2020). The most effective way to prevent damages caused by IAS is to predict their potential suitable area and take measures to limit their spread to new areas (Fournier et al., 2019). *A. adenophora* has proven to be a very aggressive invasive species in some parts of the world, including China, Australia, and South Africa. These regions have enacted costly measures to control the spread of *A. adenophora*. Therefore, it is of great significance to predict the potential suitable area patterns of *A. adenophora* under current climate conditions and future climate change scenarios.

SDMs have been widely applied to predict the potential suitable areas of IAS based on niche conservatism, which assumes that an IAS will retain a similar niche in the native and introduced regions (Ahmad et al., 2019; Graham, 2005). Although it is still controversial whether species niches are conserved across space and time (Atwater et al., 2018), recent research supports the niche conservatism hypothesis overall (Liu et al., 2020). The MaxEnt model has been widely applied in simulating species distribution (Li et al., 2019; Merow et al., 2013). In this study, we built nine MaxEnt models according to the species occurrence data and climate data under current and future scenarios (four RCPs for two time periods, 2050 and 2070) together with terrain factors, soil conditions, and land‐cover data. The predicted potential suitable area shows the same spatial pattern as the current global distribution of *A. adenophora*. To the best of our knowledge, this is the first study to model the potential suitable area of *A. adenophora* at a global scale under both current and future climate scenarios.

### 4.1 Effect of temperature change on the distribution of *A*. *adenophora*

Previous studies have shown that *A*. *adenophora* is invasive in tropical and subtropical regions, including Asia (China, India, and Nepal), Oceania (eastern Australia and New Zealand), Africa, and North America (Cronk & Fuller, 1995; Del Guacchio, 2013; Heystek et al., 2011; Kluge, 1991; Muniappan et al., 2009; Parsons et al., 2001; Tererai & Wood, 2014; Wang & Wang, 2006), and our results concur with these findings. Furthermore, we found that over 70% of the UR areas are distributed in the 36 BHRs, which are distributed in tropical and subtropical regions. Previous studies have shown that the expansion of IAS might become apparent later in invasion events and consequently have extensive negative effects on native species and the overall stability of native ecosystems(Adams & Setterfield, 2015; Mainali et al., 2015; Pyšek et al., 2012; Roger et al., 2015; Vicente et al., 2013). From this point of view, the invasion of *A. adenophora* may have serious consequences in these regions.

According to the growth environment of this weed and previous studies, the temperature is the major factor controlling the distribution of *A*. *adenophora*. A study by Wang, Lin, et al. (2017) found that the temperature during winter is the most influential factor affecting the distribution of *A*. *adenophora* in China. The research results of (Thapa et al., 2018) showed that the Minimum Temperature of Coldest Month is the most significant variable in western Himalaya. Among environmental factors, temperature, particularly the low temperature, is the main factor governing the distribution of *A. adenophora* (Li et al., 2008; Wang, Lin, et al., 2017). The above‐mentioned studies support our finding that mean temperature of coldest quarter (Bio11) was the most important factor (47.5% contribution to the model) governing the distribution of *A. adenophora*. In general, areas with warm temperatures and moist conditions are climatically suitable for invasion by *A. adenophora*, which prefers temperatures in the range of 10°C–25°C (Tererai & Wood, 2014). Thus, changes in temperature will significantly affect the distribution of *A. adenophora*. He et al. (2012) demonstrated that experimental warming increases the biomass production and canopy of *A. adenophora* and reduced mortality in comparison with its native neighbors. This means that global warming may create favorable conditions for the invasions of *A. adenophora* by promoting its growth and environmental tolerance (Poudel et al., 2019). Based on the four RCPs used in this study, global warming will continue for some decades. In theory, climatically suitable areas of *A. adenophora* in the future would expand to other regions under the background of climate warming. Chong et al. (2017) predicted that the suitable areas of *A. adenophora* would expand in Southwest China under climate warming scenarios. A similar expansion is also expected in the western Himalayas under future global warming (Lamsal et al., 2018).

However, we found that the potential suitable areas for *A. adenophora* decrease obviously on a global scale under the four RCPs in 2050 and 2070 compared with under current conditions. It may sound like a good thing because it provides opportunity for restoration of the areas which might have been currently invaded. Active restoration interventions are generally restricted by funding and thus self‐repair ability of ecosystem is expected to work. Nonetheless, is spontaneous succession a viable strategy? (Holmes et al., 2020) pointed out that the ecosystems can accomplish self‐repair under the conditions which key biotic and/or abiotic thresholds have not yet been crossed. Specifically, the identity of the invader, the ecosystem type, and the efficacy of alien control would influence this process. For example, some species can alter the soil conditions to favor its growth and release chemical drift to constrain native species (Gaertner et al., 2012; Krupek et al., 2016). This kind of “Legacy effects” would cause long‐lasting changes in ecosystem structure (D'Antonio & Meyerson, 2002; Le Maitre et al., 2011), which may lead to an alternative stable state. In this case, abiotic manipulations are required to restore the ecosystem (Le Maitre et al., 2011). Previous findings have indicated that *A. adenophora* is allelopathic (Yang, 2008; Zhong et al., 2007) and can alter soil microbial communities in its favor (Niu, Liu, & Wan, 2007; Yu et al., 2005). Furthermore, different restoration solutions are required for different ecosystems. For instance, lowland fynbos ecosystems are said to be less resilient to invasion and have a lower capacity for self‐repair compared with mountain fynbos ecosystems (Holmes et al., 2020). This means that active restoration is necessary for these areas of low self‐repair capacity. Anyway, large capital costs are required for restoration, thus preventing invasions early is vastly preferable.

### 4.2 Whether *A*. *adenophora* will shift toward higher elevation under future climate change scenarios?

Under global warming, some species will migrate to higher latitudes or higher elevations to adapt to climate change (Bertrand et al., 2011; Hackett et al., 2008; Root et al., 2003), especially in mountain ecosystems (Felde et al., 2012). Under current climate conditions, the distribution of *A. adenophora* with respect to elevation is similar in native and introduced regions. *A. adenophora* is distributed in areas with elevations ranging from 520 to 3,200 m in its native range (Mexico) (Sang et al., 2010), while it is found at elevations between 330 and 2,500 m in China (Wang & Wang, 2006) and between 400 and 3,280 m in Nepal (Shrestha et al., 2018). According to Sunil et al. (2018), *A. adenophora* is expected to move to elevations up to 3,547 m a. s. l. by 2070. Wang and Wang (2006) explored the characteristics of the invasion process in China during different periods and found that the upper elevation limit of the species distribution increased from 1,800 m before 1960 to 2,500 m during 1991–2003. Our results show that the spatial pattern and altitudinal distribution of this weed change under future climate change scenarios. In the altitude range of 500–1,500 m, UR areas decreased under the eight RCPs, while the opposite trend was observed for elevations exceeding 1,500 m. No matter from the view of potential suitable areas or percentages at different elevation ranges, the potential suitable area of *A. adenophora* would expand in elevation ranges between 3,000 and 3,500 m. In combination with a decreasing trend globally, a likely explanation is that *A. adenophora* will shift upslope under future climate conditions and thus face consistent reductions in the area that this species can occupy (Liang et al., 2018). Though previous studies have indicated that the species toward higher elevations or latitudes is predicted to increase with climate change, most of the evidences were observed from the occurrence records collected from the fields (Dainese et al., 2017; Kelly & Goulden, 2008; Steinbauer et al., 2018; Vanderwal et al., 2013). We predicted the expansion of *A. adenophora* at higher elevation ranges, which could not figure out the drivers of this kind of expansion (from lower area or not).

Biological invasions are considered to be the 5th important impact of human activities on the earth's environment (Brondizio et al., 2019). Montane ecosystems, which have high biodiversity and are sensitive to climate change, are of particular concern under climate warming (Dullinger et al., 2012). Among terrestrial ecosystems, mountain ecosystems and particularly high mountains are often considered to be at low risk of invasion (Pauchard et al., 2009). However, the invasion process is driven by a combination of climate change and human activities (Alexander et al., 2016). Increasing anthropogenic activities offer more opportunities for the invasion of non‐native species, and road networks are regarded as the major pathway for IAS invasion. There will be at least 25 million kilometers of new roads anticipated by 2050, with developing countries accounting for 90% of this increase (Laurance et al., 2014). This will provide opportunities for the establishment of non‐native species and conduits for their dispersal (Becker et al., 2005); roads and trails are recognized as major pathways for invasion into mountains (Fuentes et al., 2010; Lembrechts et al., 2014; Pauchard & Alaback, 2004). Hence, a detailed assessment of the effects of road infrastructure on biodiversity is needed given the rapid expansion of road networks.

### 4.3 Uncertainty

The limitations of this study can be summarized as follows. Since MaxEnt is an ecological niche model, only the abiotic factors were taken into consideration (Ahmad et al., 2019; Xu et al., 2019). As indicated by the “BAM” (abiotic factors, biotic factors, and movement) diagram (Pauchard & Alaback, 2004), the distribution of a species is governed not only by abiotic factors but also by biotic factors including interactions between species and dispersal ability. It should also be noted that we only used MaxEnt model in this research instead of using an ensemble model, some research found that ensembles outperform individual models (Crossman & Bass, 2008; Marmion et al., 2009). In this study, the land‐cover conditions along with climate variables were used as input to the model; however, we assumed that the land‐cover conditions would remain unchanged in the future. Climate factors were considered to be the principal factors in other global‐ or country‐scale studies of species distribution. To better understand the influence of climate change on species distribution, the intraspecific interactions and changes in land cover should be taken into consideration. Furthermore, the current climate conditions in this study are not “current” for the current climate data derived from interpolations of observed data (representative of 1960–2000). During the past two decades, the world climate has changed greatly, which may affect the accuracy of the model (Wang et al., 2018). The newly released CMIP6 applied a new set of emissions scenarios, shared socioeconomic pathways (SSPs; O'Neill et al., 2017), is said to make future scenarios more reasonable and thus more reliable than before (Di Luca et al., 2020; Nie et al., 2020; Su et al., 2021). Finally, although we have determined the regions of native occurrence from all records, the intentional introduction of *A. adenophora* was not taken into consideration. This may explain why the occurrence of *A. adenophora* is always near urban/built‐up regions.

## 5 CONCLUSIONS

Detecting the potential suitable regions for species invasion is of great significance for preventing IAS invasion. Based on the MaxEnt model, the potential invasion ranges of *A. adenophora* under current and future climate conditions were evaluated. Our results show that the potential invasion range of *A. adenophora* is mainly distributed in subtropical and warmer temperate regions, including southwestern America, Chile, the Himalayas, southwestern China, and southeastern Australia. Among environmental factors, the mean temperature of coldest quarter contributes the most to the model, and the optimal temperature range for this species is 8°C–16°C. Although the invasion range of *A. adenophora* will shrink globally under all RCPs, the invasion risk will increase in six biodiversity hotspot regions (BHRs), such as Mountains of Southwest China, with a clear expansion trend at higher elevations under future climate scenarios. The findings provide reference information for developing appropriate management strategies to prevent the establishment and further spread of *A. adenophora* across the globe, especially in BHRs. Research findings in our study call for special concern on biological invasions in BHRs, especially in mountain regions.

## CONFLICT OF INTEREST

The authors declare that they have no conflict of interest.

## AUTHOR CONTRIBUTIONS

**Gu Changjun:** Data curation (lead); Formal analysis (lead); Methodology (lead); Software (lead); Writing‐original draft (lead). **Tu Yanli:** Funding acquisition (equal); Supervision (supporting); Writing‐review & editing (supporting). **Liu Linshan:** Conceptualization (lead); Funding acquisition (supporting); Investigation (lead); Methodology (supporting); Supervision (lead); Visualization (supporting); Writing‐review & editing (lead). **Wei Bo:** Data curation (supporting); Formal analysis (supporting); Supervision (supporting); Visualization (lead). **Zhang Yili:** Funding acquisition (lead); Methodology (supporting); Supervision (equal); Writing‐review & editing (equal). **Yu Haibin:** Methodology (equal); Supervision (supporting); Validation (supporting). **Wang Xilong:** Funding acquisition (supporting); Supervision (supporting). **Yangjin Zhuoga:** Investigation (supporting); Resources (supporting). **Zhang Binghua:** Supervision (supporting); Visualization (supporting); Writing‐review & editing (supporting). **Cui Bohao:** Supervision (supporting); Visualization (supporting).

## Supporting information

Figures S1‐S3

Tables S1‐S8

## Data Availability

Data on the spatial distribution of *Ageratina adenophora* occurrence, potential suitable area of A. adenophora under eight RCPs, distributions of *A. adenophora* UR regions in different elevation ranges within the Mountains of Southwest China BHR are available from datadryad, and distributions of *A. adenophora* UR regions in the Himalayas under current conditions and a future climate change scenario (RCP8.5 2070; https://datadryad.org/stash/share/gl0QaDZj9T8dRdTuNJvrj2dt0G9ZZvsdhsNueE07Wt8).
