# Supplementary material for: Predicting the potential global distribution of Ageratina adenophora under current and future climate change scenarios
Source: Ecol Evol. 2021 Aug 8;11(17):12092–113. doi: 10.1002/ece3.7974 (PMC8427655; doi:10.1002/ece3.7974)
Supplement: Supplementary file 1 — Figures S1‐S3 [file ECE3-11-12092-s001.docx]

# Predicting the potential global distribution of *Ageratina adenophora* under current and future climate change scenarios

Gu Changjun *et al*: Predicting the potential global distribution of *Ageratina adenophora* under current and future climate change scenarios

Gu Changjun^1,2^, Tu Yanli^3^，Liu Linshan^1 *^，Wei Bo^1,2^, Zhang Yili^1,2,4^, Yu Haibin^5^, Wang Xilong^3^, Yangjin Zhuoga^3^, Zhang Binghua^1,2^, Cui Bohao^1,2^

^1^ Key Laboratory of Land Surface Pattern and Simulation, Institute of Geographic Sciences and Natural Resources Research, CAS, Beijing 100101;

^2^ University of Chinese Academy of Sciences, Beijing 100049, China;

^3^ Tibet Plateau Institute of Biology, Lhasa 850000, China;

^4^ CAS Center for Excellence in Tibetan Plateau Earth Sciences, Beijing 100101, China.

^5^ School of Life Sciences, Guangzhou University, Guangzhou 510006, China;

***** Correspondence: [liuls@igsnrr.ac.cn](mailto:liuls@igsnrr.ac.cn)


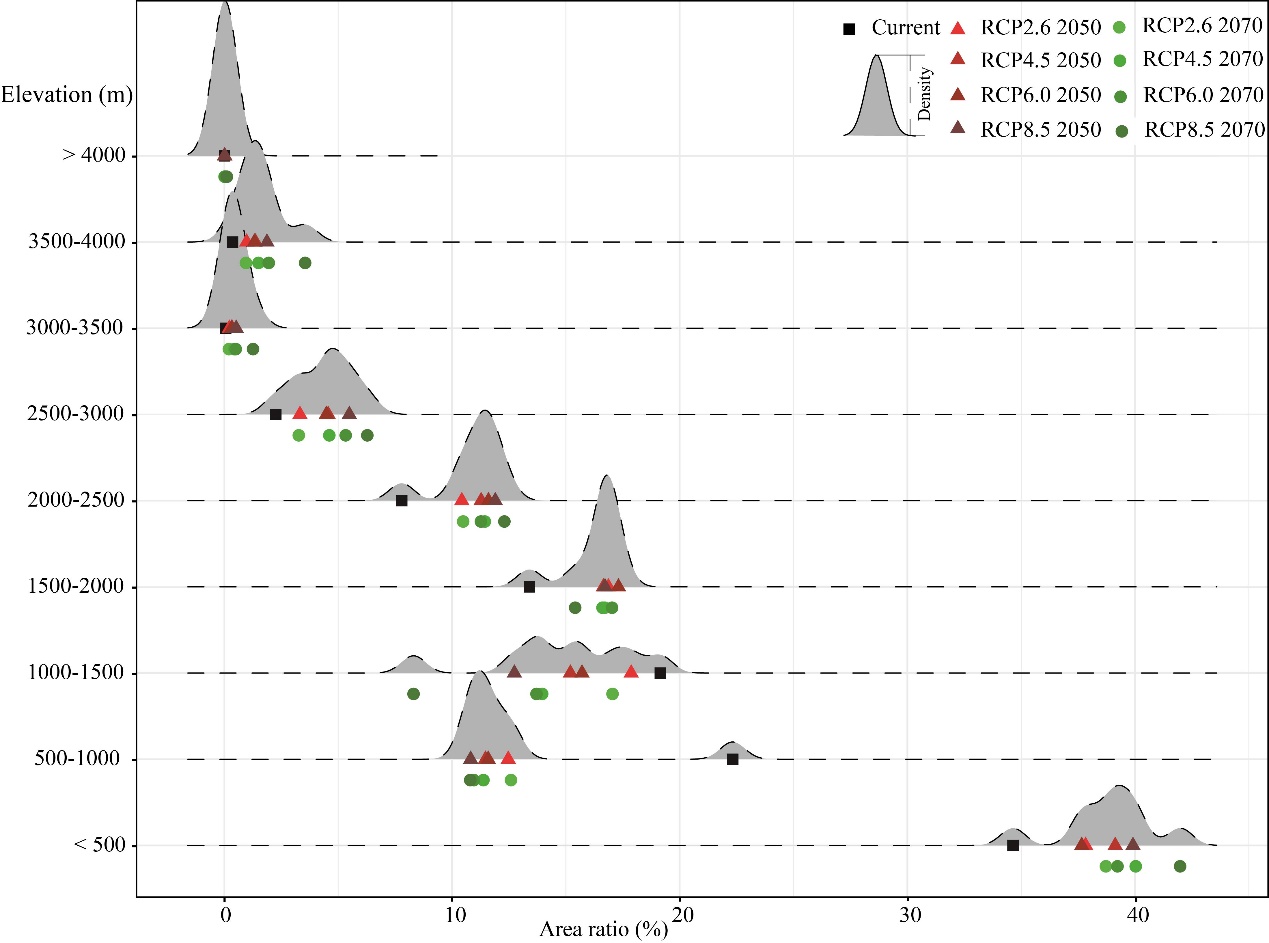


Figure S1. Distributions of MR regions for *A. adenophora* within different elevation ranges. Red triangles denote the four RCPs in 2050, while the green dots represent the four RCPs in 2070. To improve the visibility of differences between the RCPs for 2050 and 2070, the four RCPs for 2070 are located beneath the four RCPs for 2050.


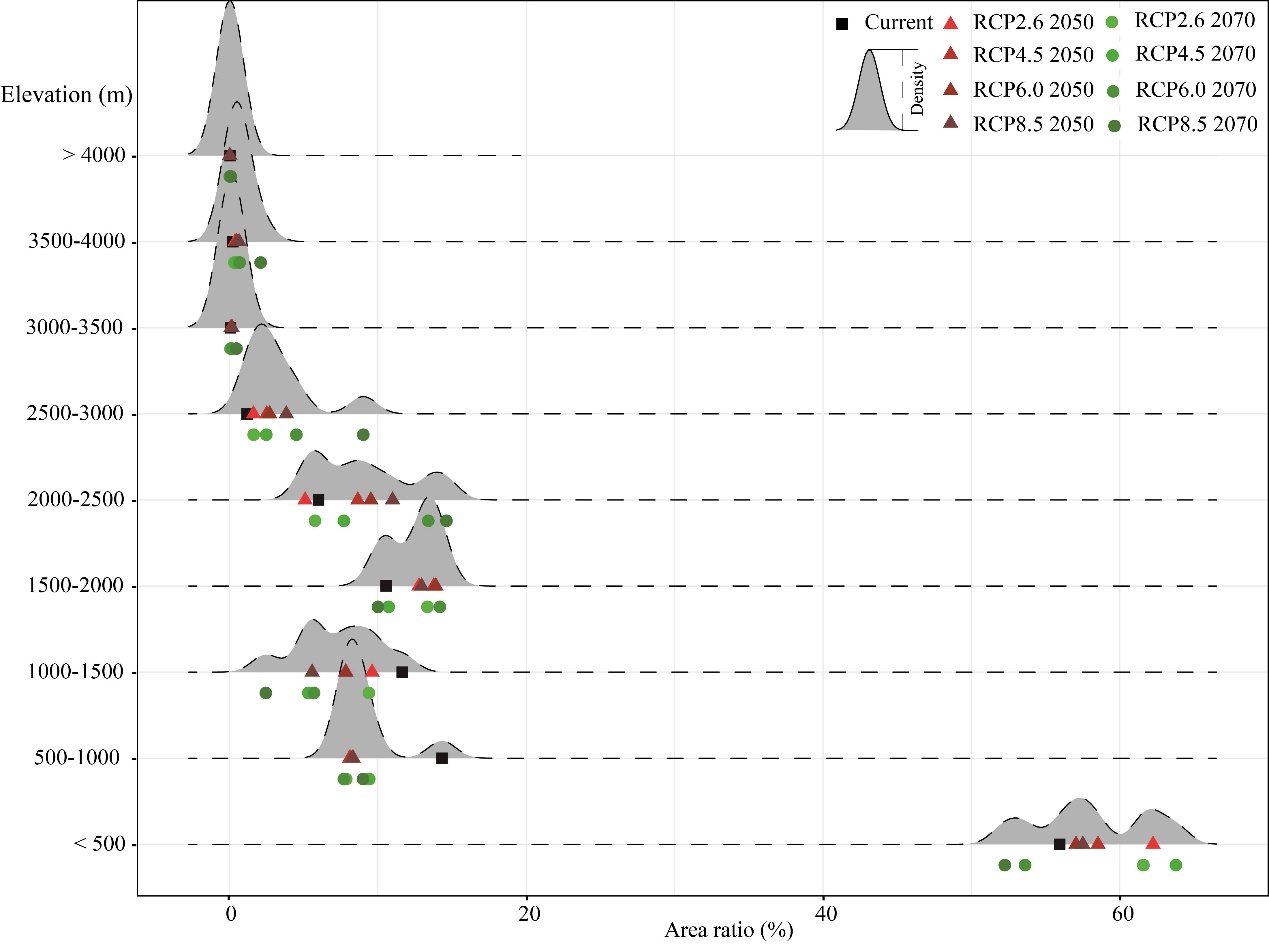


Figure S2. Distributions of HR regions for *A. adenophora* within different elevation ranges. Red triangles denote the four RCPs in 2050, while the green dots represent the four RCPs in 2070. To improve the visibility of differences between the RCPs for 2050 and 2070, the four RCPs for 2070 are located beneath the four RCPs for 2050.


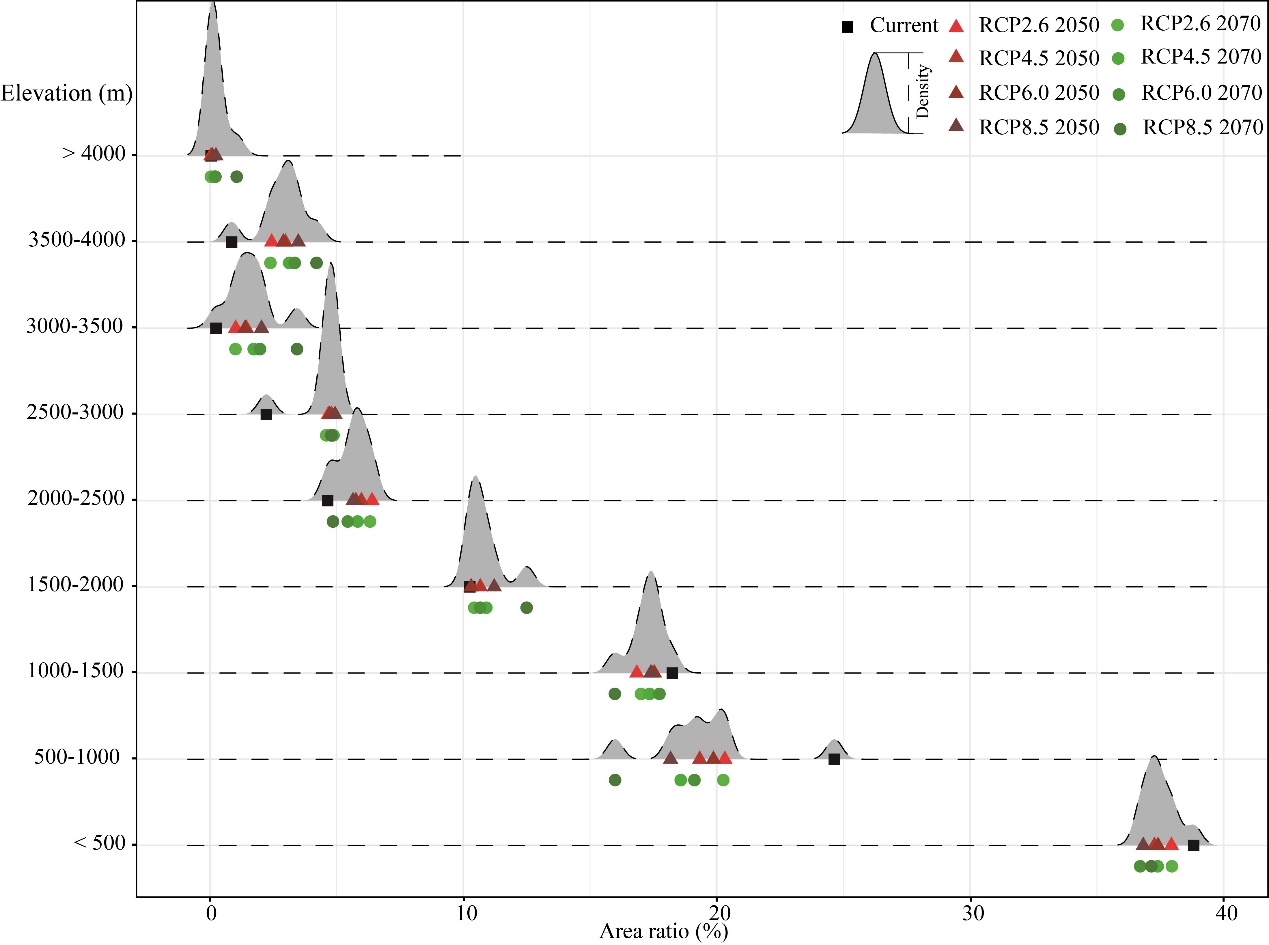


Figure S3. Distributions of LR regions for *A. adenophora* within different elevation ranges. Red triangles denote the four RCPs in 2050, while the green dots represent the four RCPs in 2070. To improve the visibility of differences between the RCPs for 2050 and 2070, the four RCPs for 2070 are located beneath the four RCPs for 2050.
